# Supplementary material for: Minority-centric meta-analyses of blood lipid levels identify novel loci in the Population Architecture using Genomics and Epidemiology (PAGE) study
Source: PLoS Genet. 2020 Mar 30;16(3):e1008684. doi: 10.1371/journal.pgen.1008684 (PMC7145272; doi:10.1371/journal.pgen.1008684)
Supplement: S1 Fig — (A) HDL for the minority meta-analysis; (B) LDL for the minority meta-analysis; (C) TC for the minority meta-analysis; (D) TG for the minority meta-analysis; (E) TC for the Hispanic-specific meta-analysis; (F) HDL for the minority plus European meta-analysis; (G) LDL for the minority plus European meta-analysis; (H) TC for the minority plus European meta-analysis; (I) TG for the minority plus European meta-analysis. (DOCX) [file pgen.1008684.s001.docx]

**Supplementary Fig.1 QQ plots of the meta-analyses. (A) HDL for the minority meta-analysis; (B) LDL for the minority meta-analysis; (C) TC for the minority meta-analysis; (D) TG for the minority meta-analysis; (E) TC for the Hispanic-specific meta-analysis; (F) HDL for the minority plus European meta-analysis; (G) LDL for the minority plus European meta-analysis; (H) TC for the minority plus European meta-analysis; (I) TG for the minority plus European meta-analysis.**

(A) (B)

**
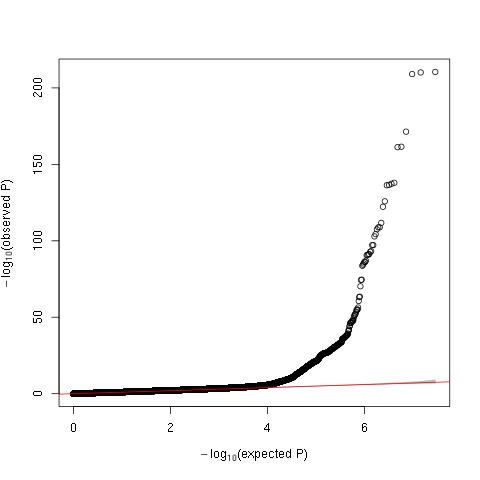

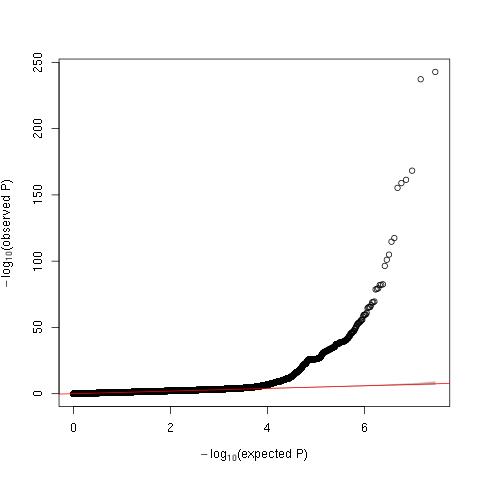
**(C) (D)

λ=1.057

λ=1.066

**
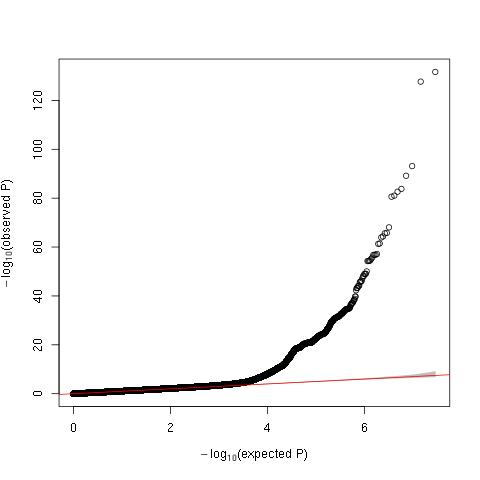

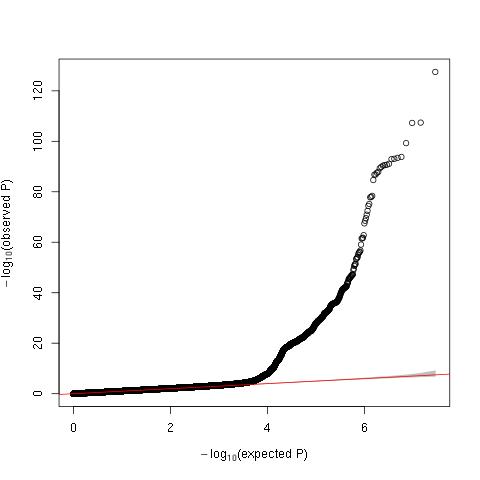
**

λ=1.046

λ=1.061

(E) (F)

**
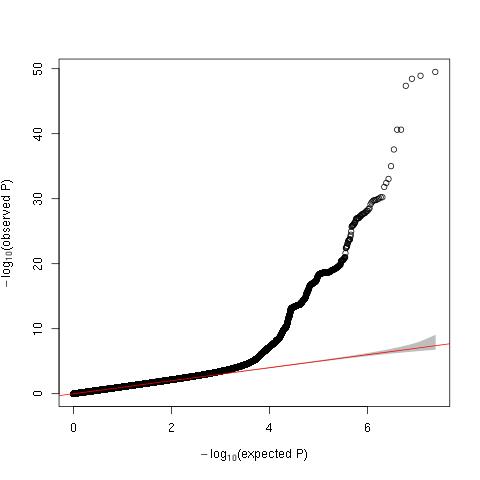

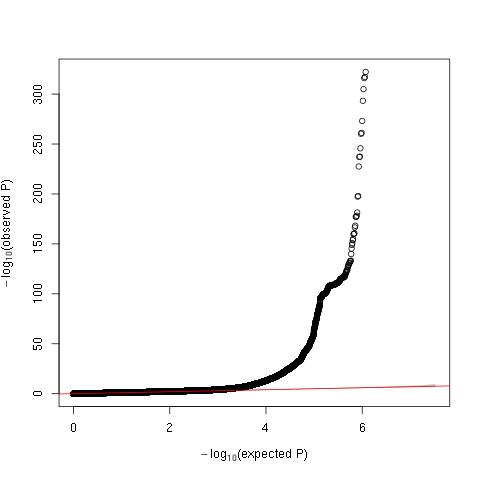
**

λ=1.085

λ=1.066

(G) (H)

**
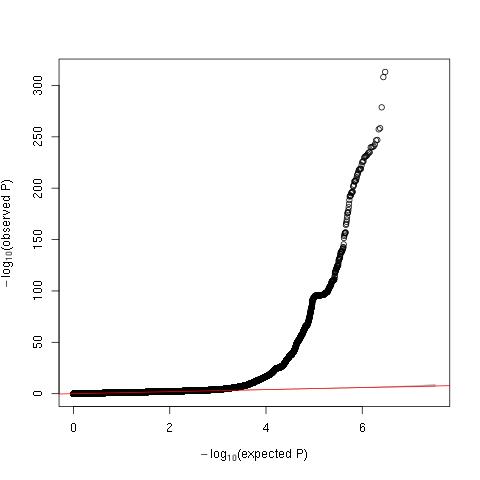
**
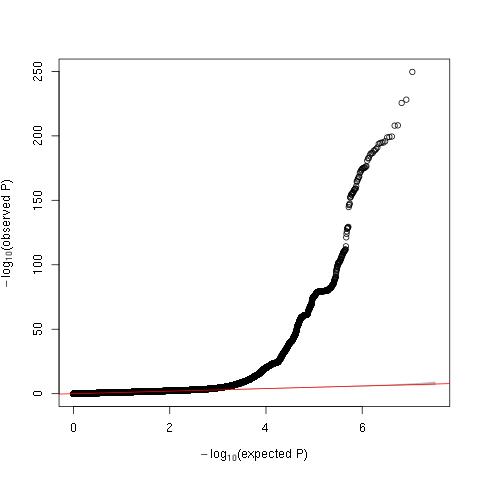


λ=1.075

λ=1.071

(I)


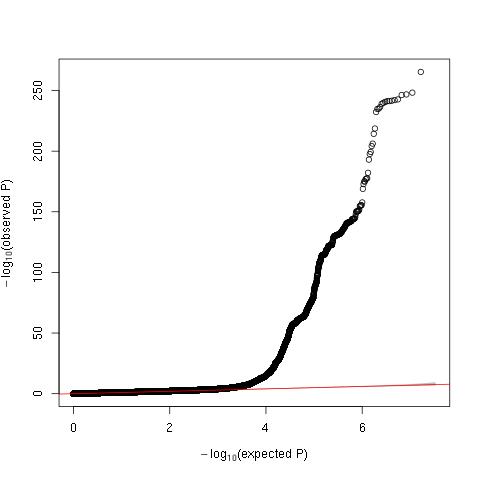


λ=1.066
