# Supplementary material for: Minority-centric meta-analyses of blood lipid levels identify novel loci in the Population Architecture using Genomics and Epidemiology (PAGE) study
Source: PLoS Genet. 2020 Mar 30;16(3):e1008684. doi: 10.1371/journal.pgen.1008684 (PMC7145272; doi:10.1371/journal.pgen.1008684)

**Supplementary Fig. 2 Locuszoom plots for the nine novel loci. Genetic coordinates are displayed along the x-axis (hg19) and genome-wide association significance level is plotted against the y-axis as -log_10_(*P* value). LD is indicated by color scale in relationship to the most significant SNP (colored as purple diamond) in each association (red: r^2^≥0.8, orange: 0.6≤r^2^<0.8, green: 0.4≤r^2^<0.6, blue: 0.2≤r^2^<0.4, navy: r^2^<0.2). (A) *5q31* for HDL; (B) *DLC1* for HDL; (C) *ZCCHC6* for HDL; (D) *DDHD1* for HDL; (E) *HLF* for HDL; (F) *B4GALNT3* for LDL; (G) *GPCPD1* for LDL; (H) *PCSK1* for TC; (I) *B4GALNT3* for TC; (J) *GPCPD1* for TC; (K) *MTHFD2* for TG.**

(A)


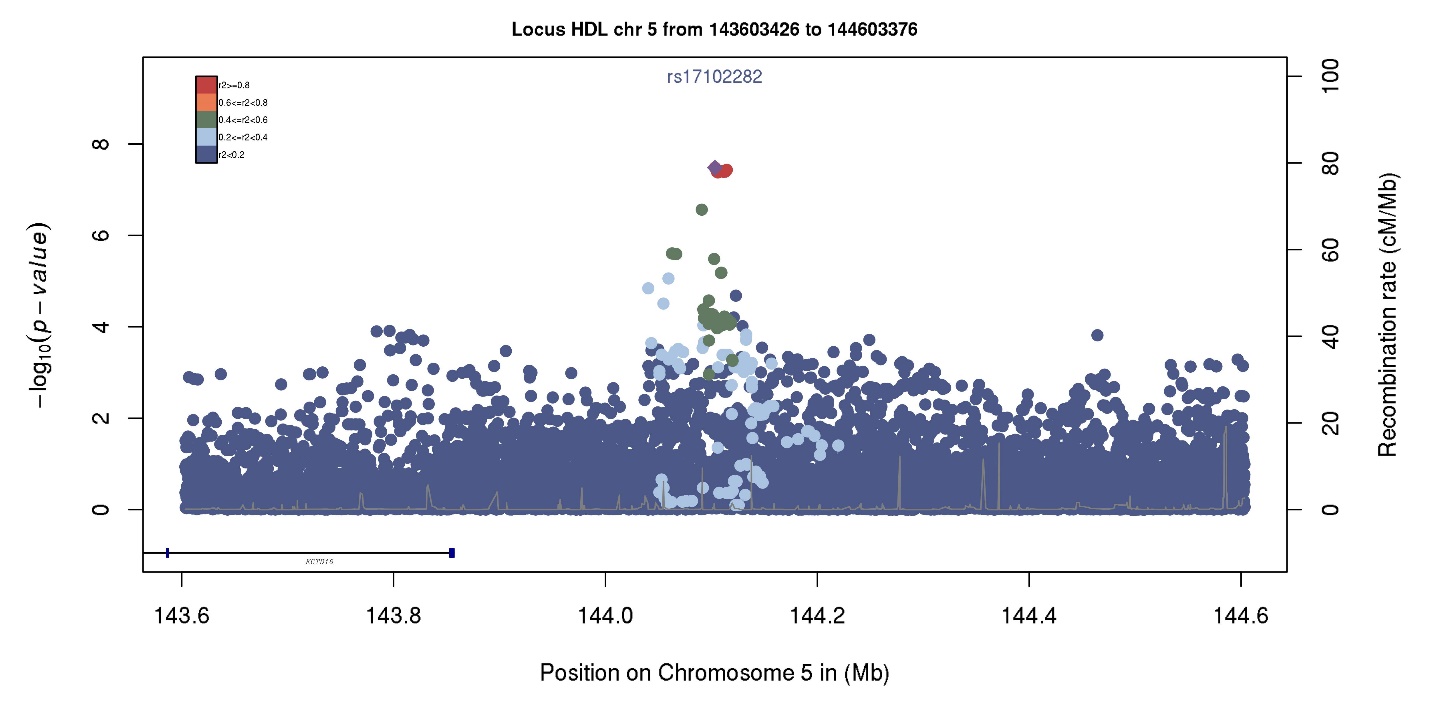


(B)


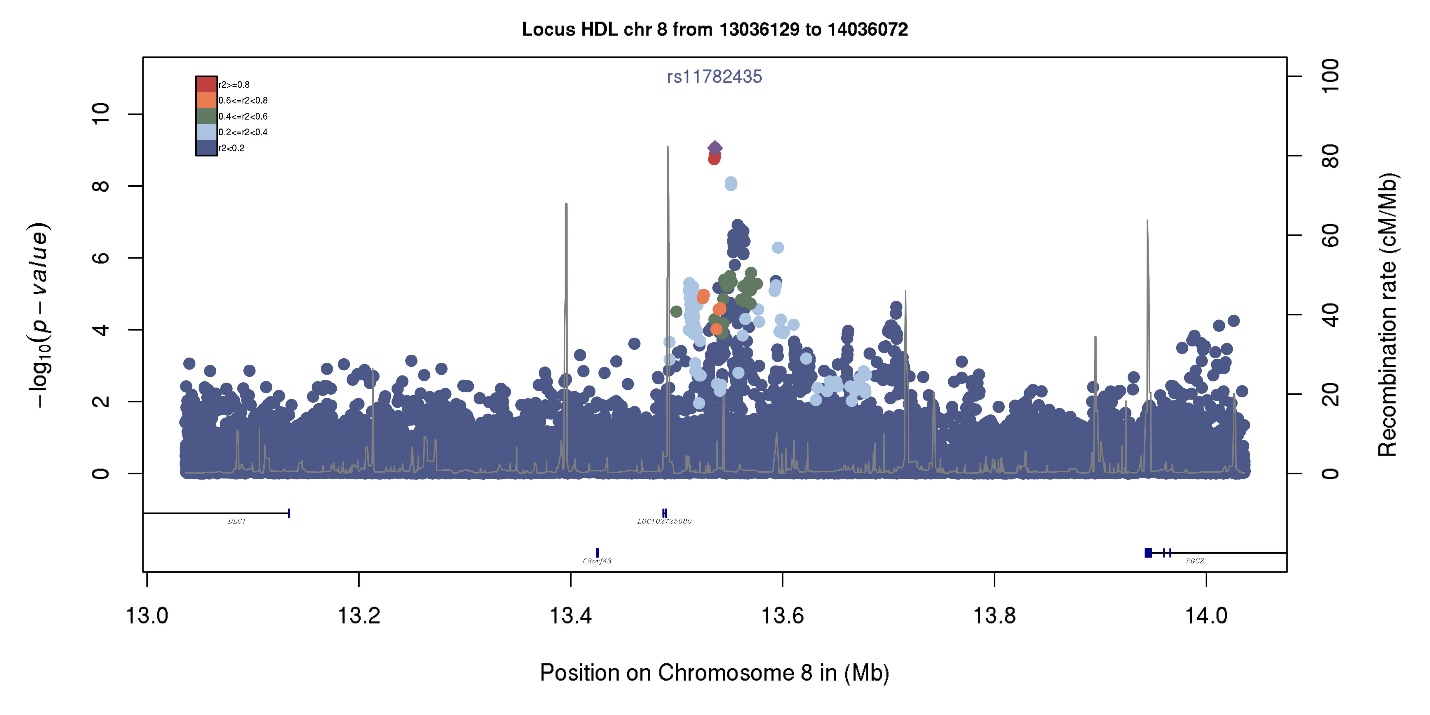


(C)


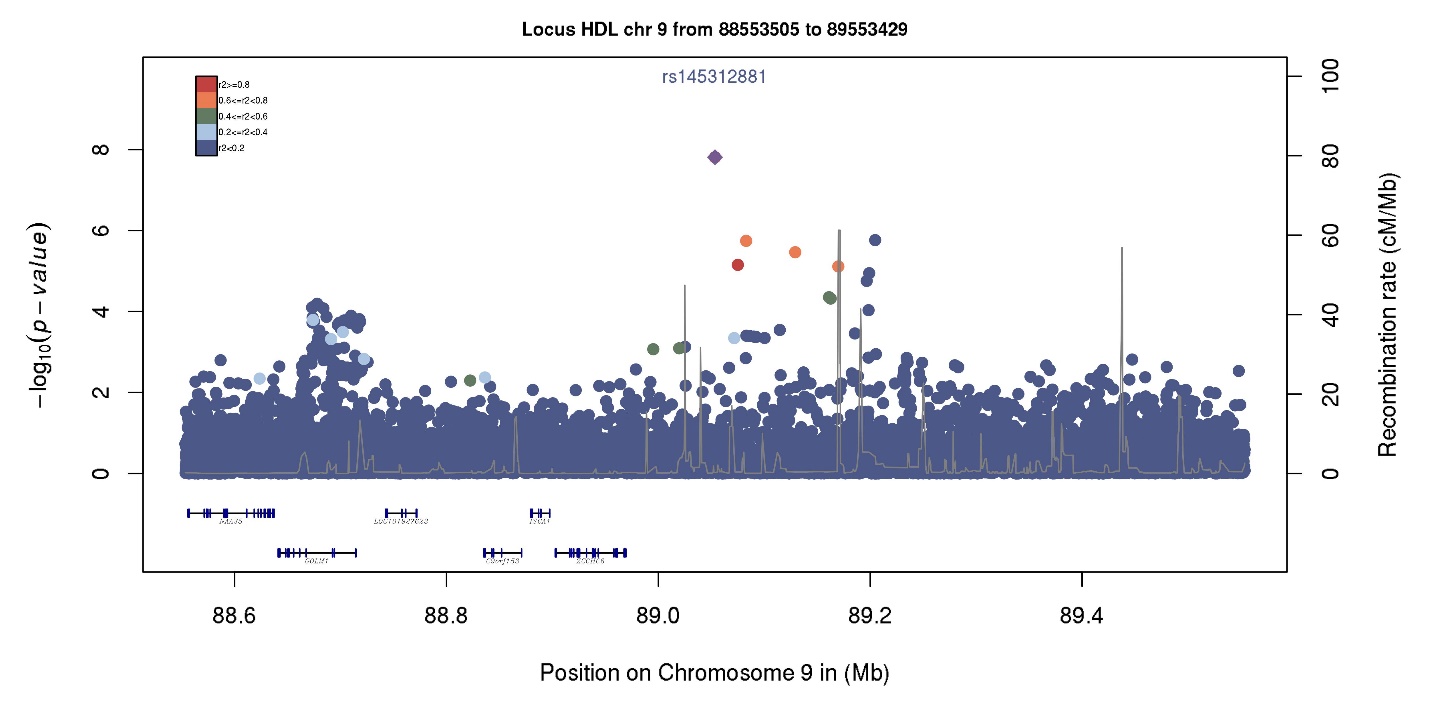


(D)
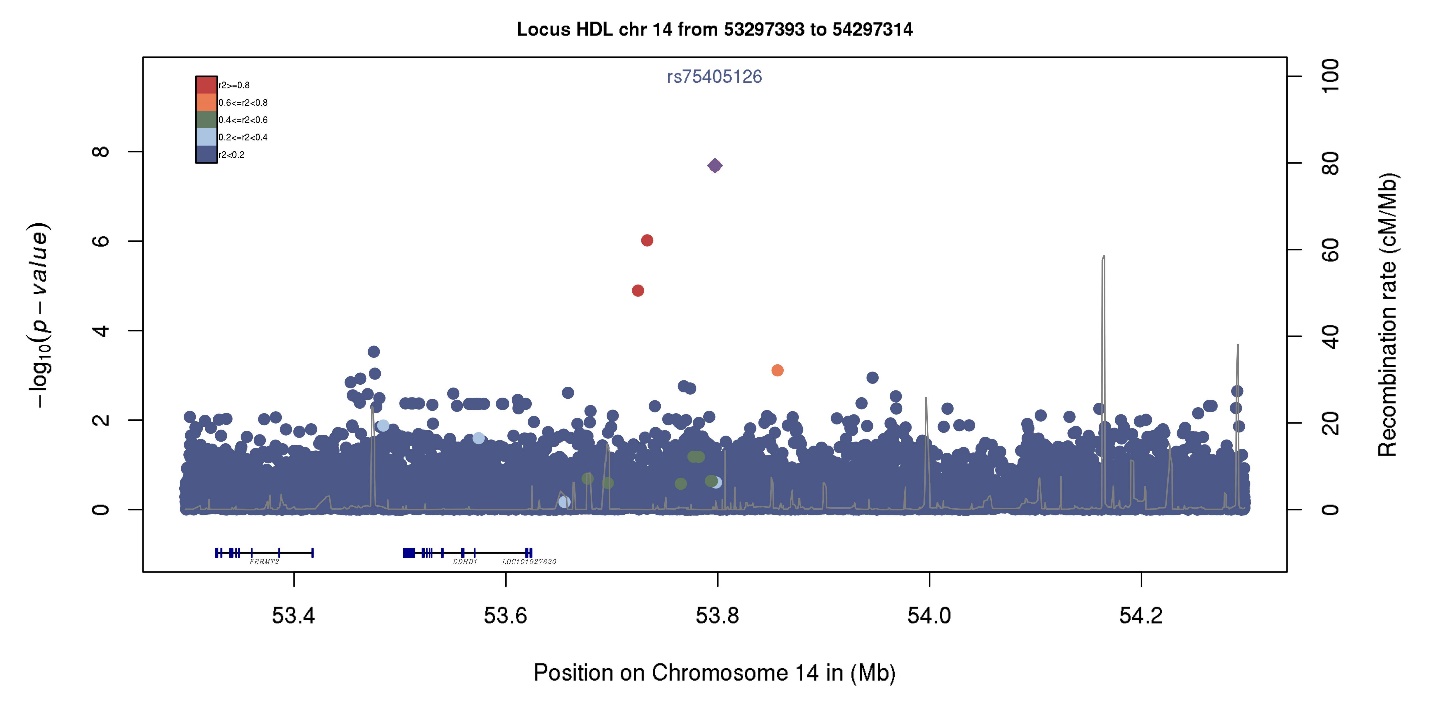


(E)


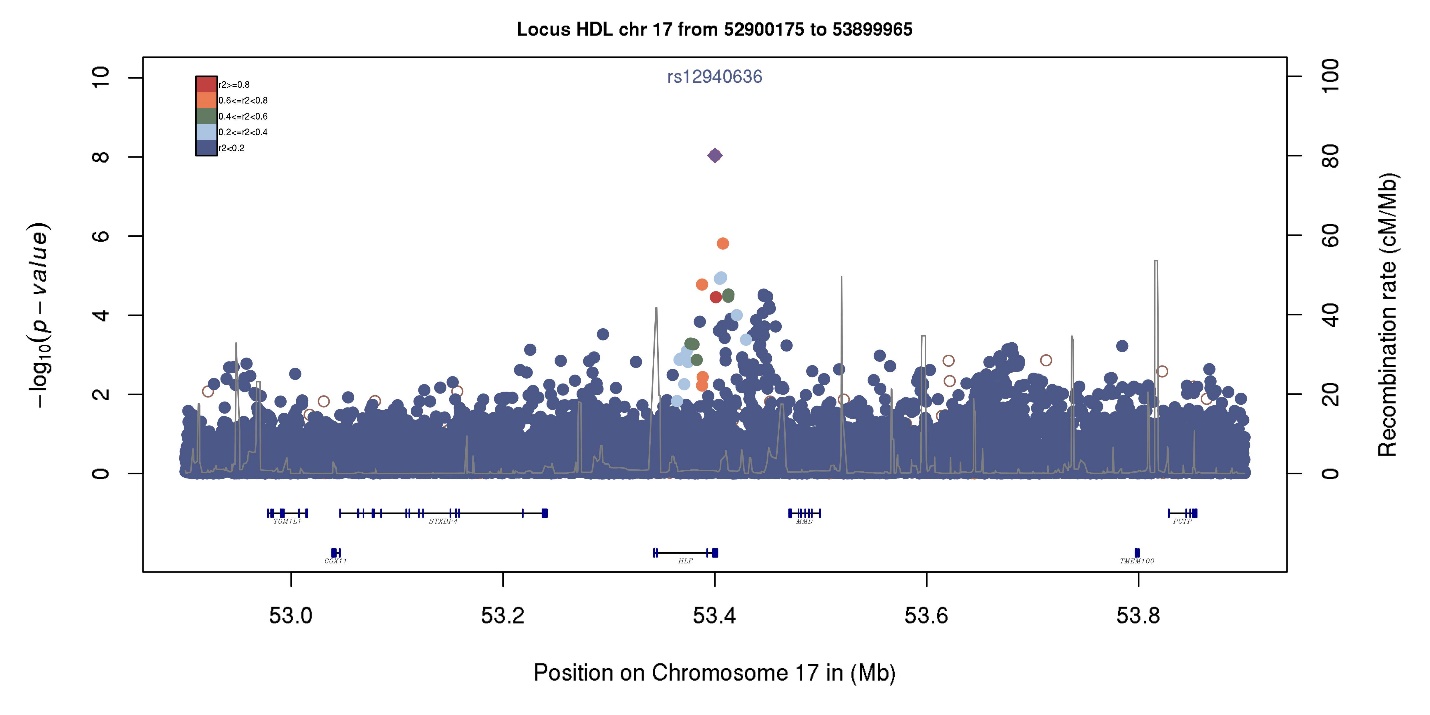


(F)


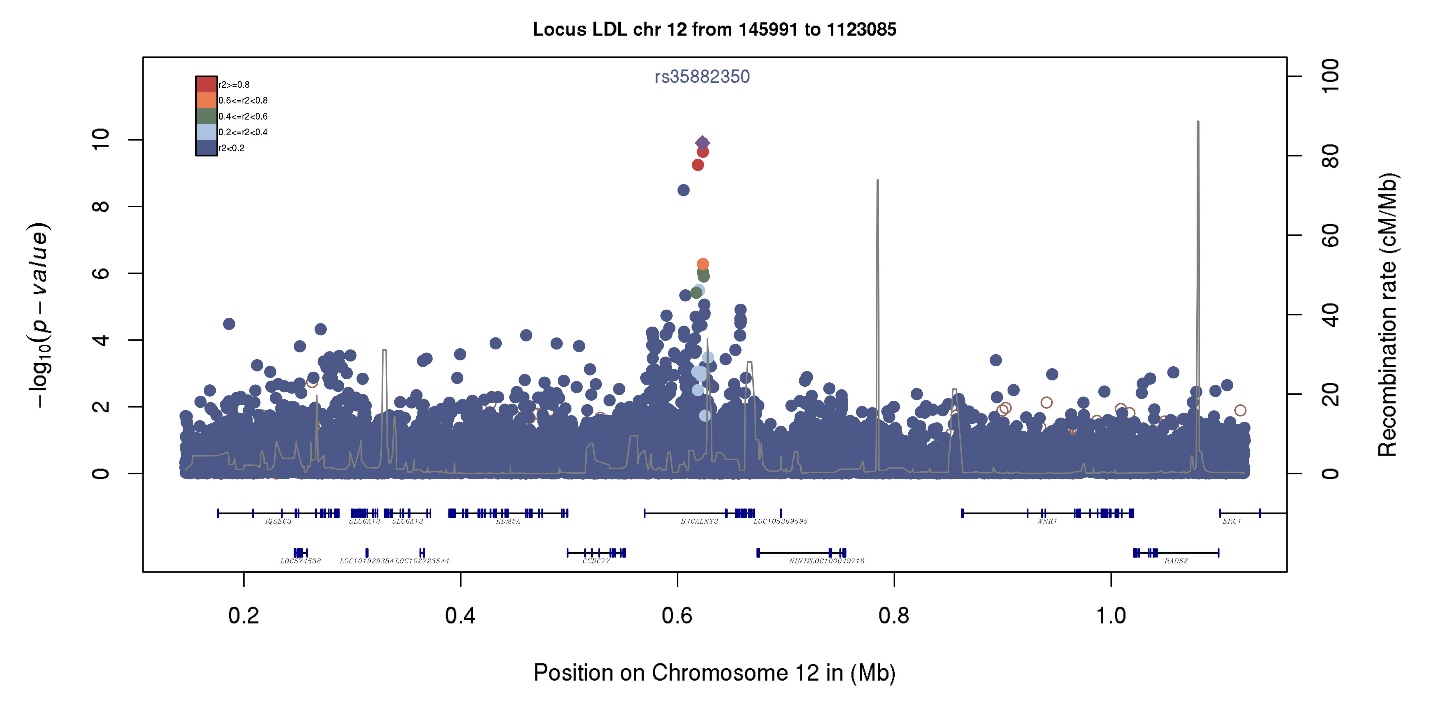


(G)


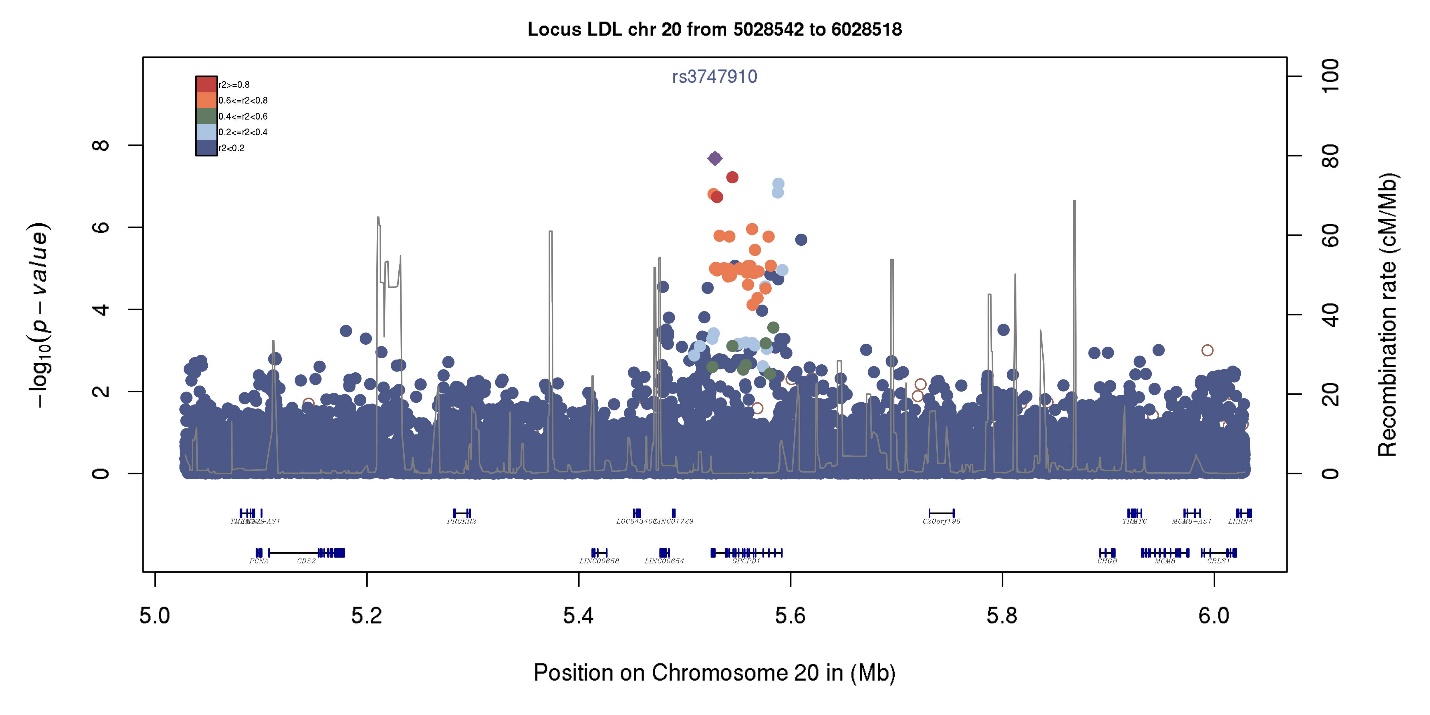


(H)


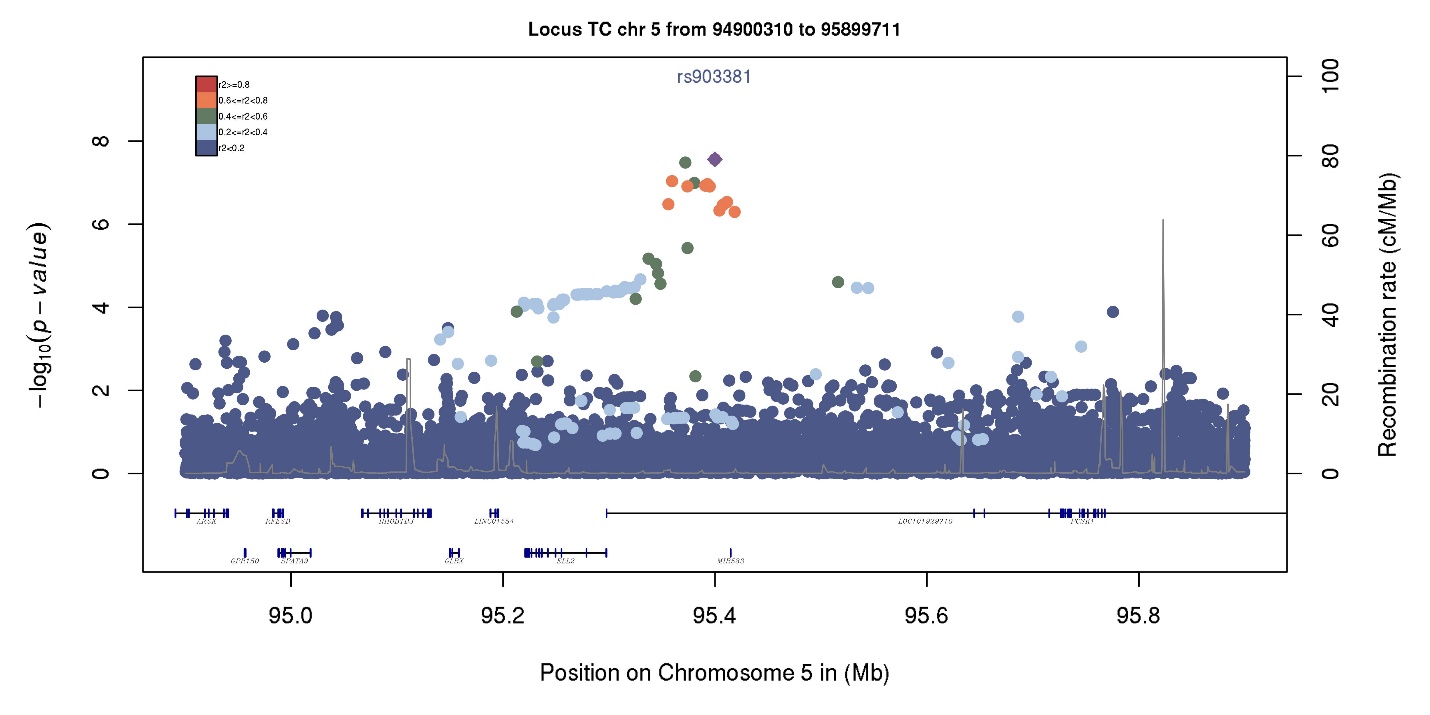


(I)


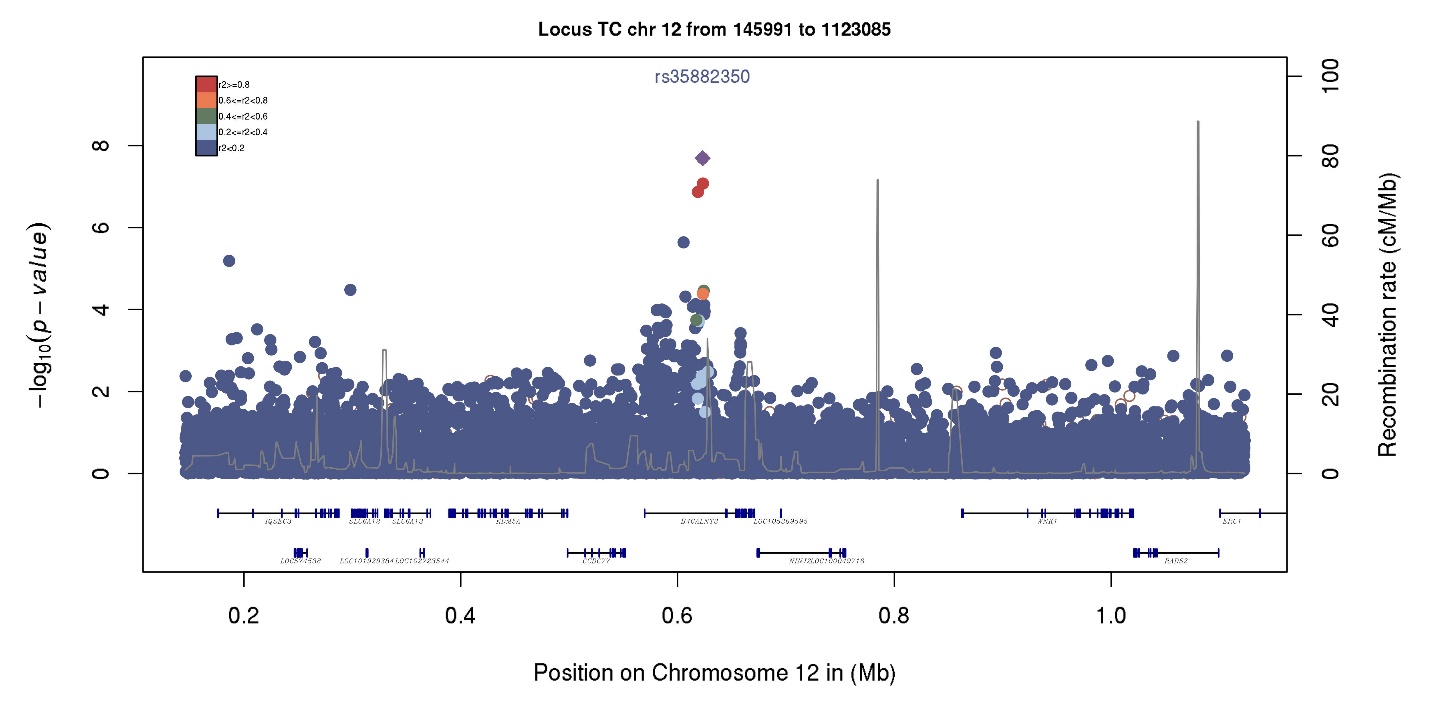


(J)


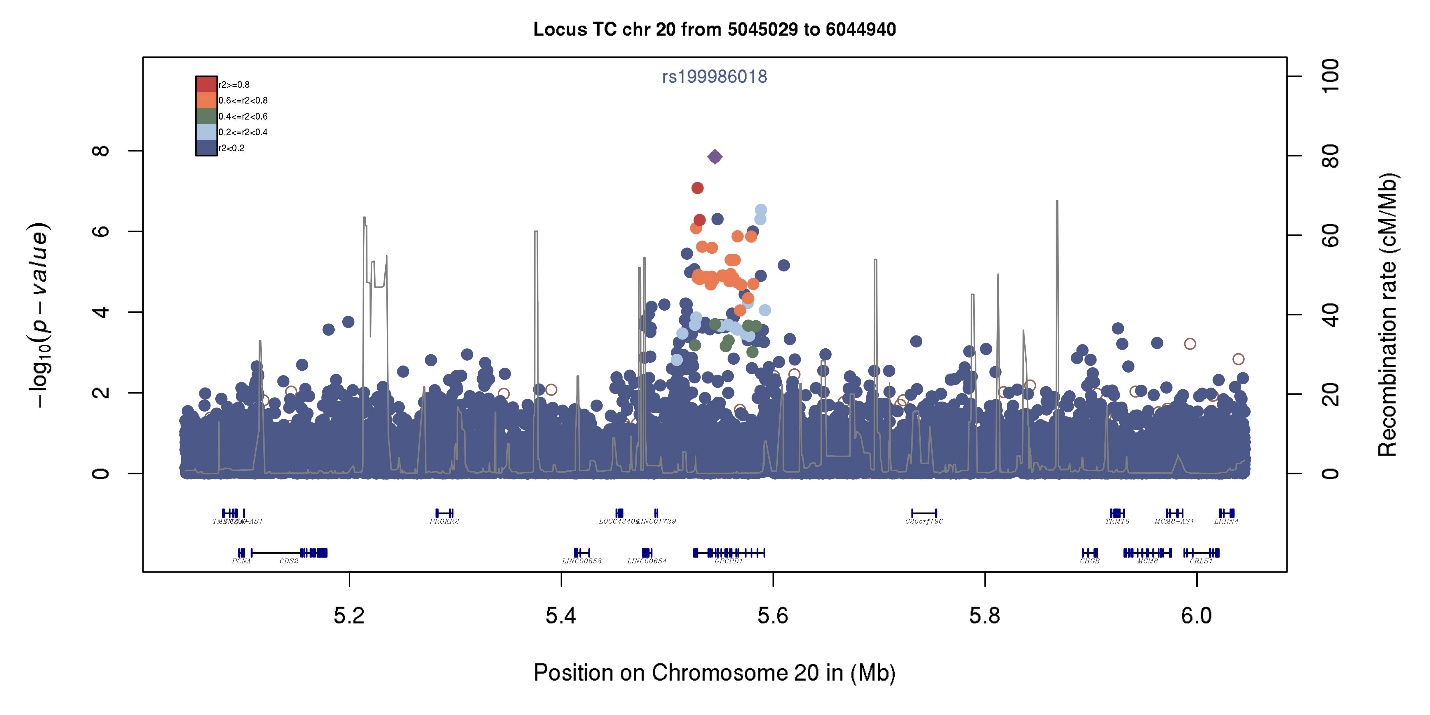


(K)


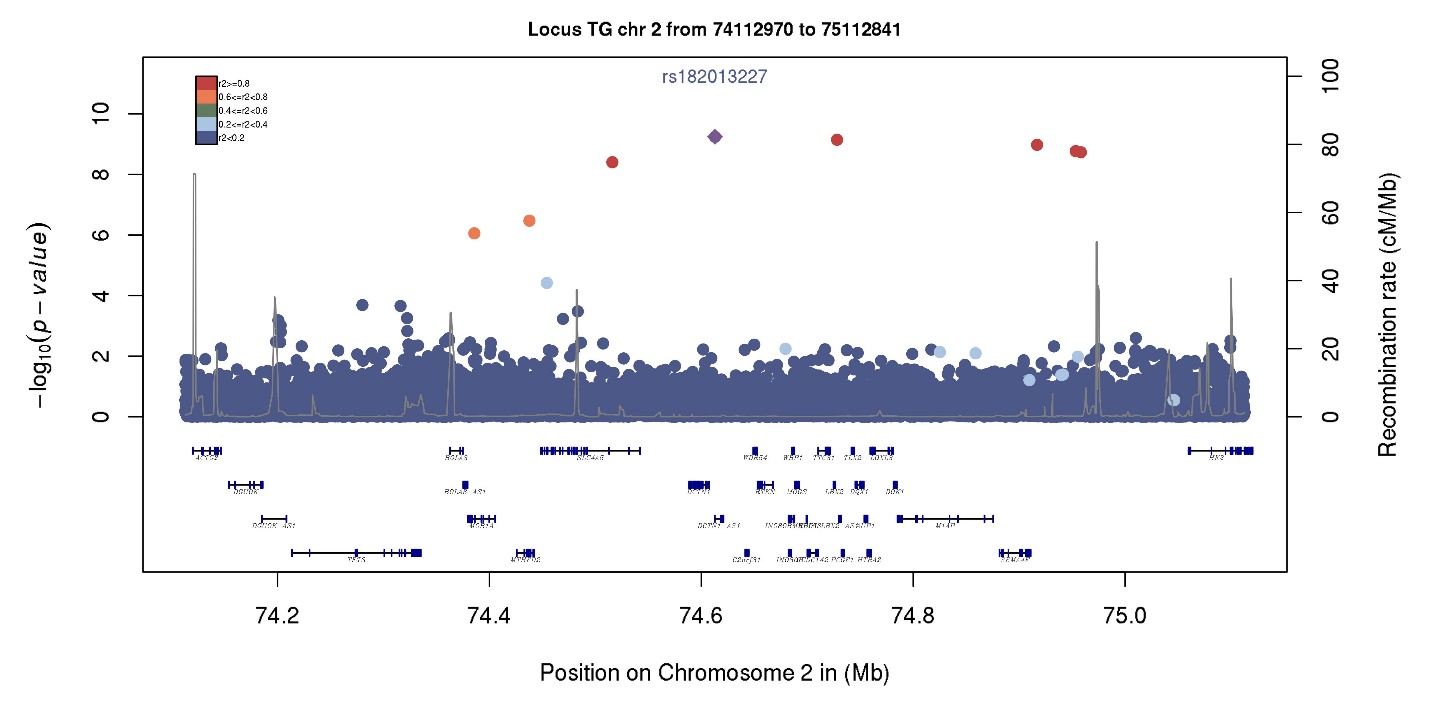

Supplement: S2 Fig — Genetic coordinates are displayed along the x-axis (hg19) and genome-wide association significance level is plotted against the y-axis as -log10(P value). LD is indicated by color scale in relationship to the most significant SNP (colored as purple diamond) in each association (red: r2≥0.8, orange: 0.6≤r2<0.8, green: 0.4≤r2<0.6, blue: 0.2≤r2<0.4, navy: r2<0.2). (A) 5q31 for HDL; (B) DLC1 for HDL; (C) ZCCHC6 for HDL; (D) DDHD1 for HDL; (E) HLF for HDL; (F) B4GALNT3 for LDL; (G) GPCPD1 for LDL; (H) PCSK1 for TC; (I) B4GALNT3 for TC; (J) GPCPD1 for TC; (K) MTHFD2 for TG. (DOCX) [file pgen.1008684.s002.docx]
