# Supplementary material for: Minority-centric meta-analyses of blood lipid levels identify novel loci in the Population Architecture using Genomics and Epidemiology (PAGE) study
Source: PLoS Genet. 2020 Mar 30;16(3):e1008684. doi: 10.1371/journal.pgen.1008684 (PMC7145272; doi:10.1371/journal.pgen.1008684)

**Supplementary Fig. 3 Functional annotation of the nine novel loci. The top hit at each locus was colored purple, and the SNPs showing r^2^≥0.8, 0.8>r^2^≥0.6, 0.6>r^2^≥0.4 and 0.4>r^2^≥0.2 were colored red, orange, green and blue, respectively. (A) *5q31*; (B) *DLC1*; (C) *ZCCHC6*; (D) *DDHD1*; (E) *HLF*; (F) *B4GALNT3*; (G) *GPCPD1*; (H) *PCSK1*; (I) *MTHFD2*.**

(A)

**
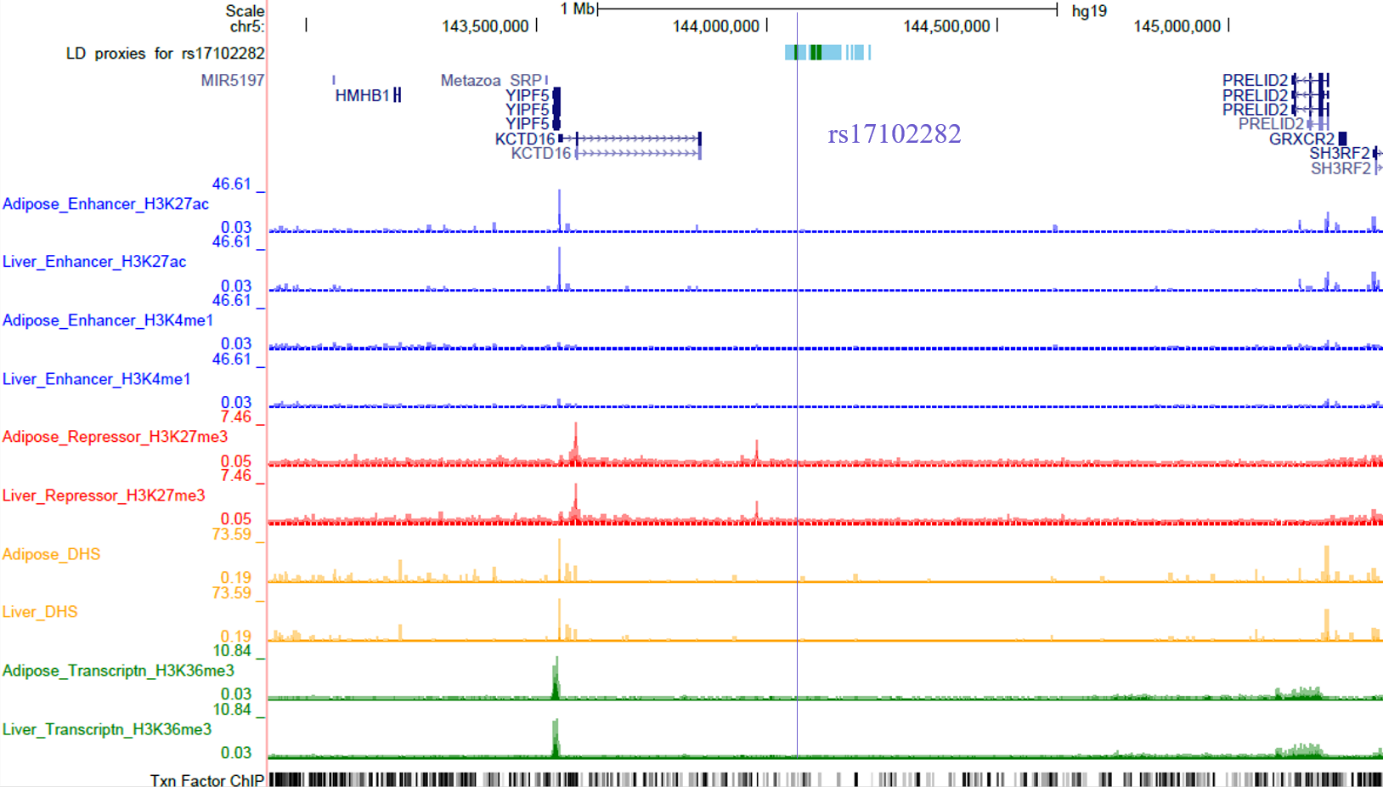
**

(B)


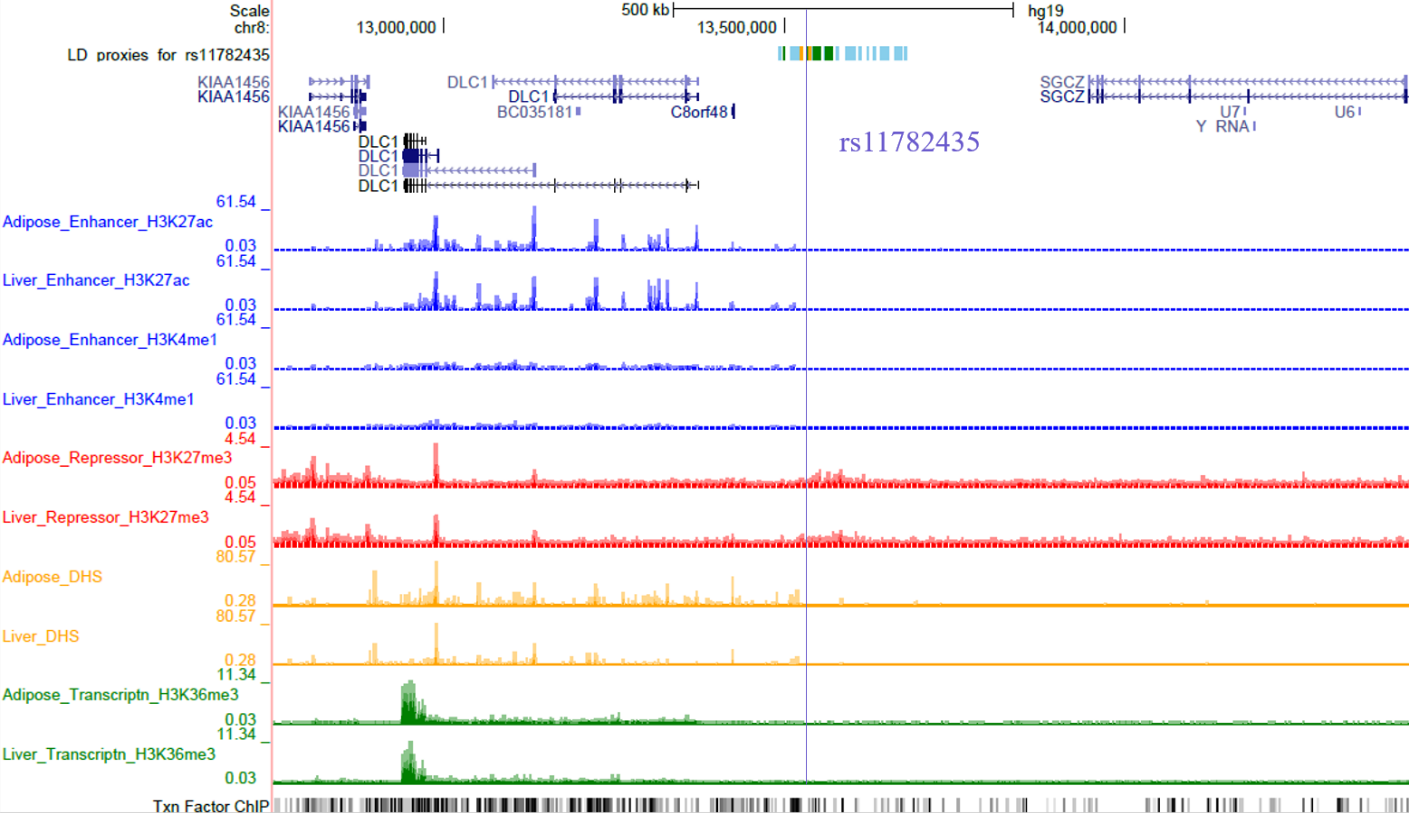


(C)


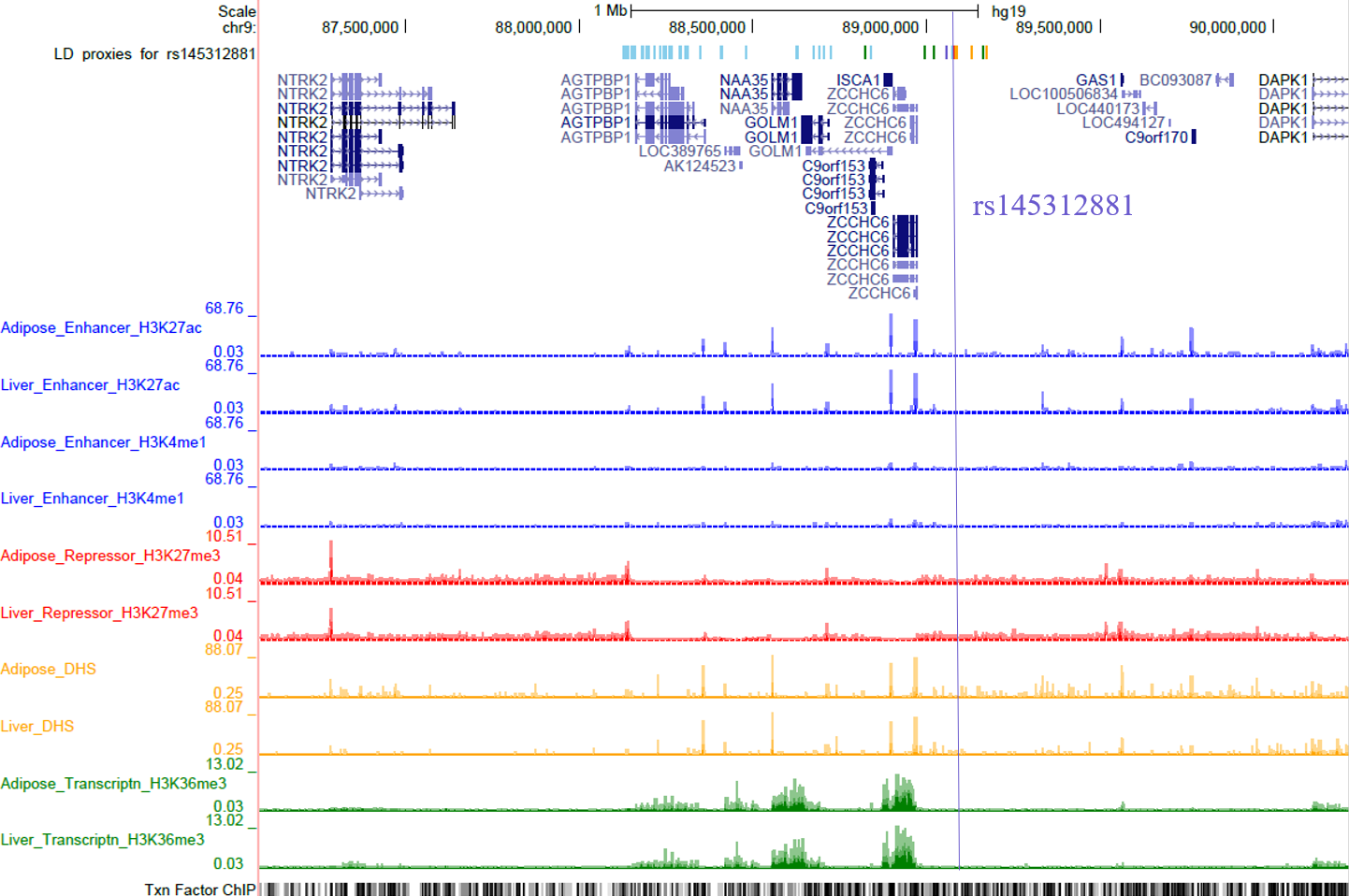


(D)


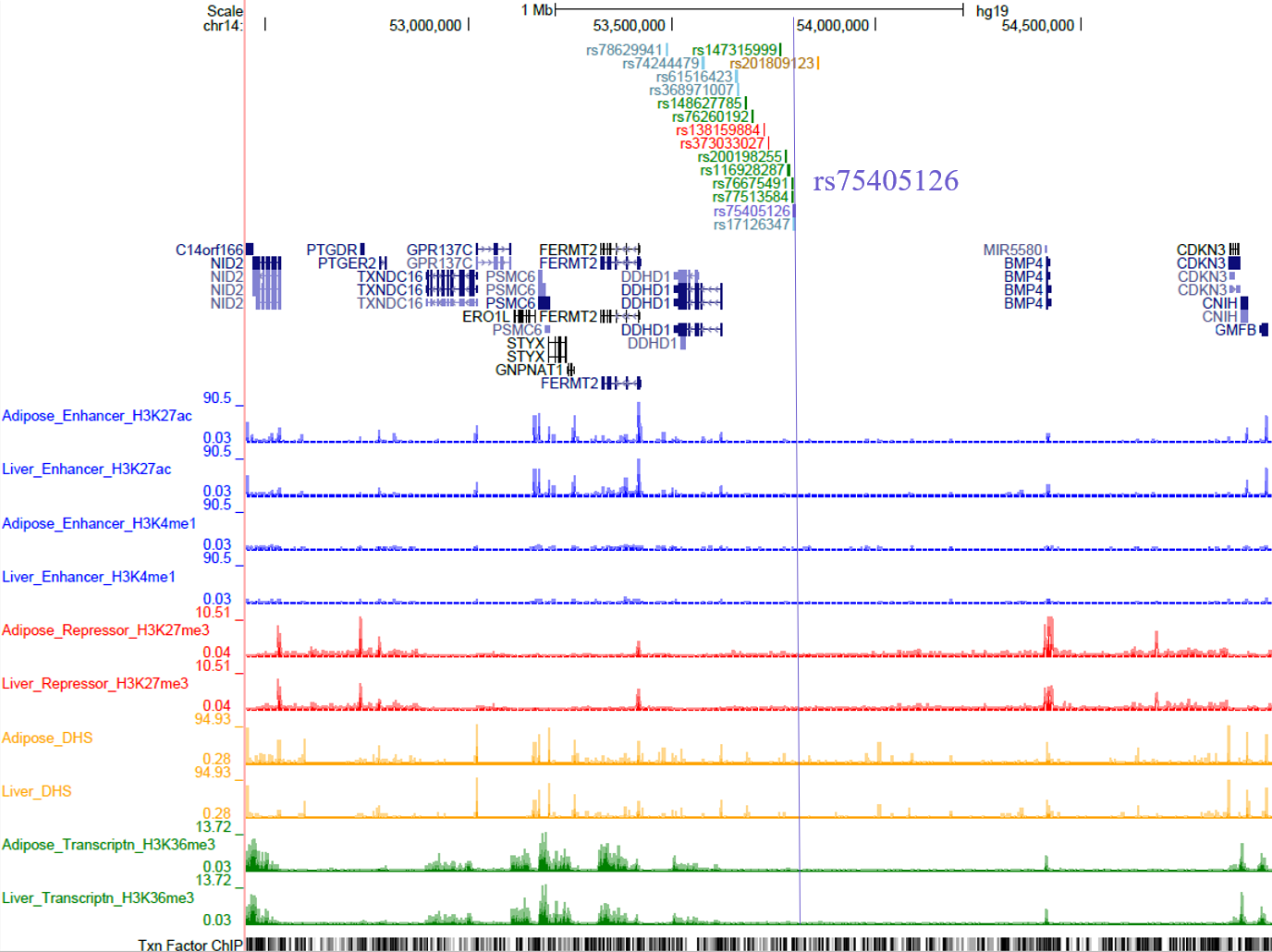


(E)


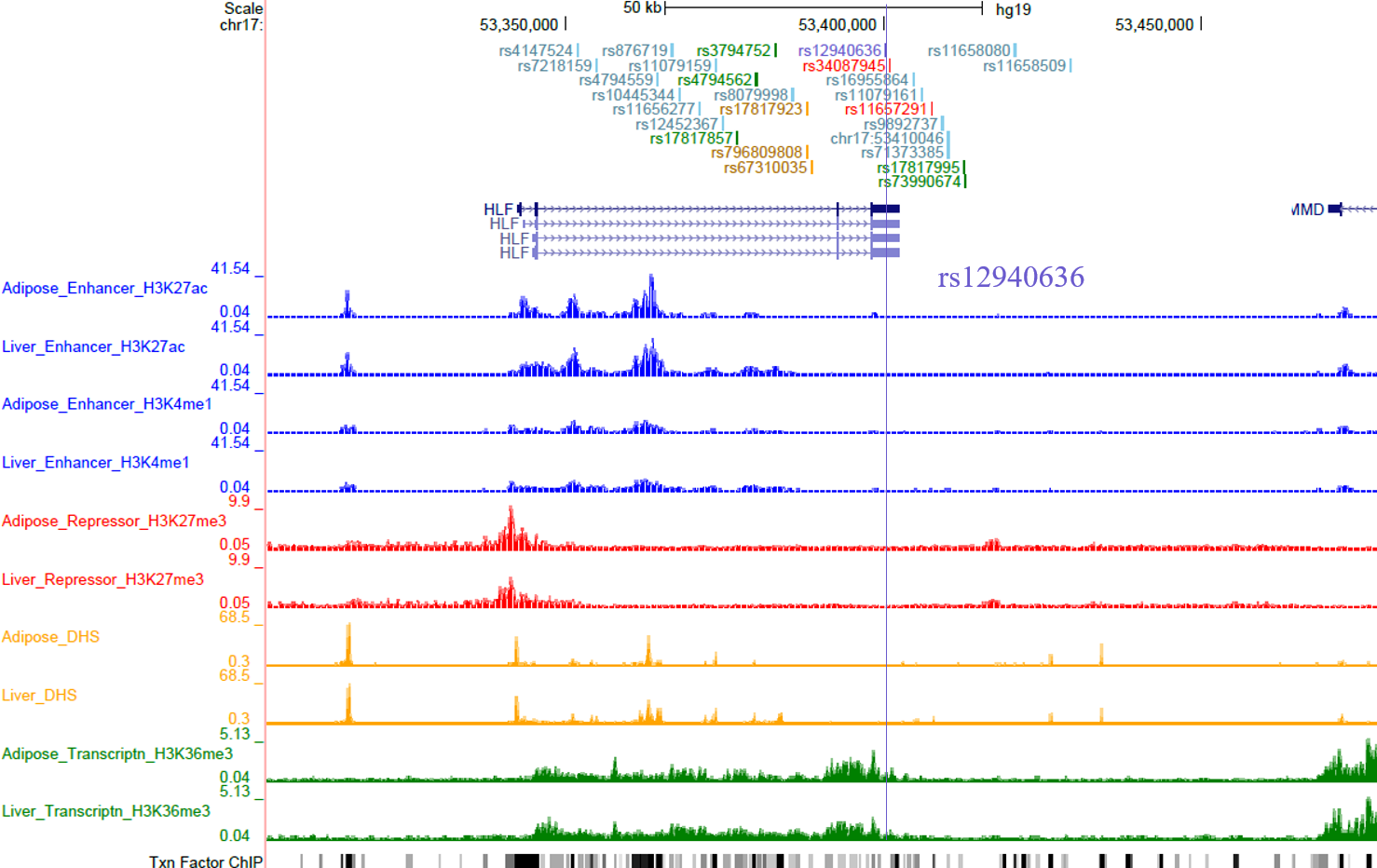


(F)


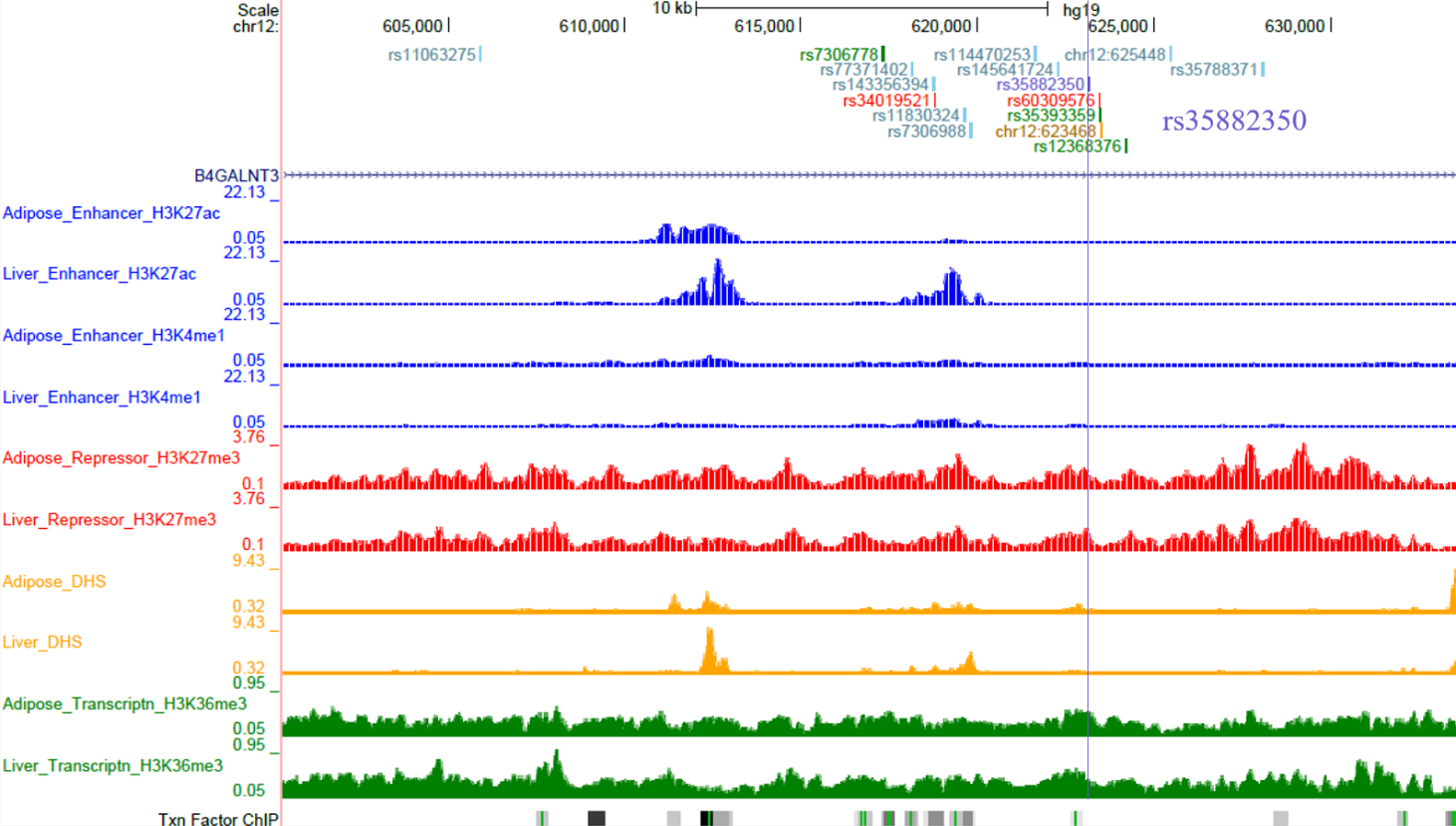


(G)


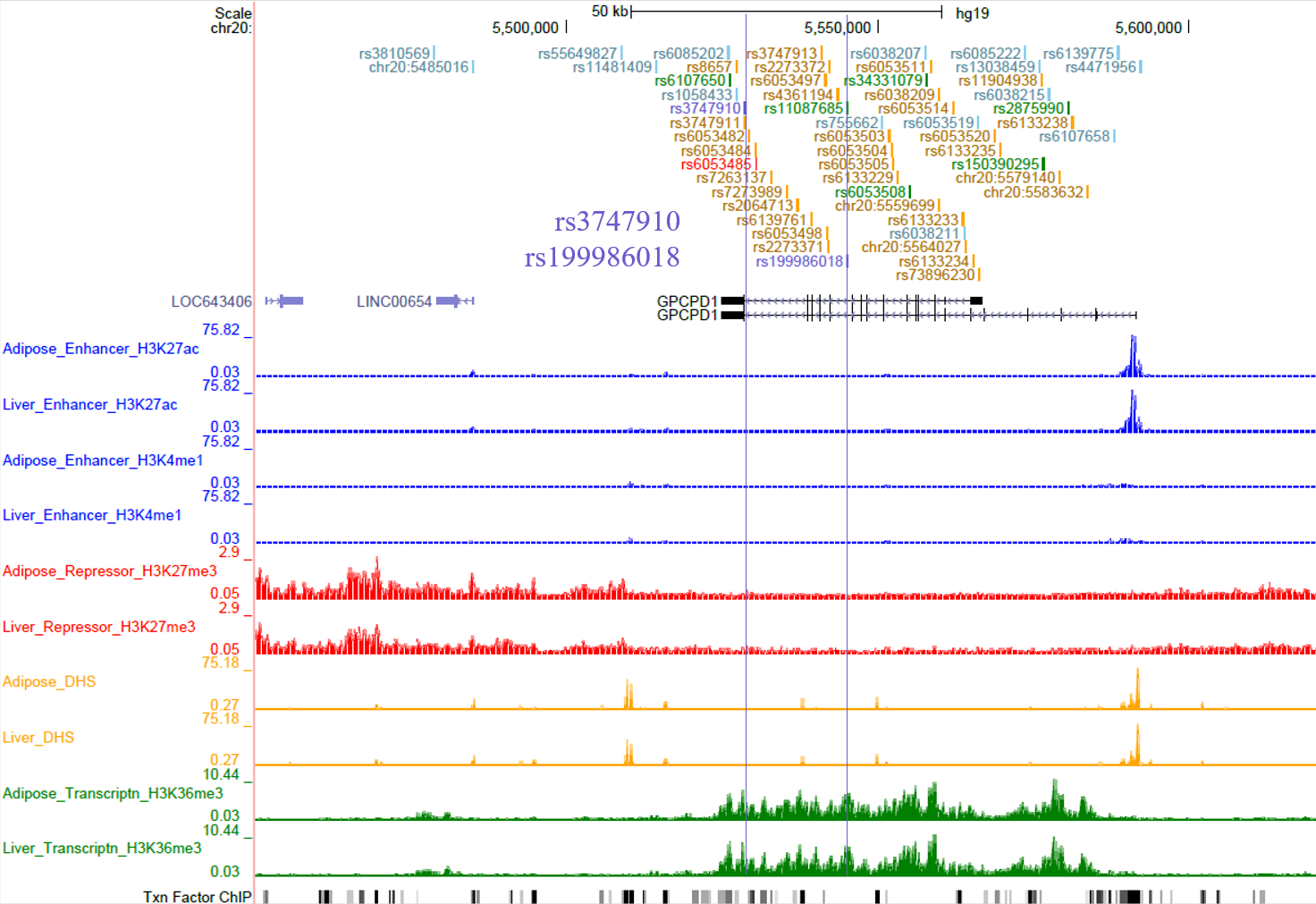


(H)


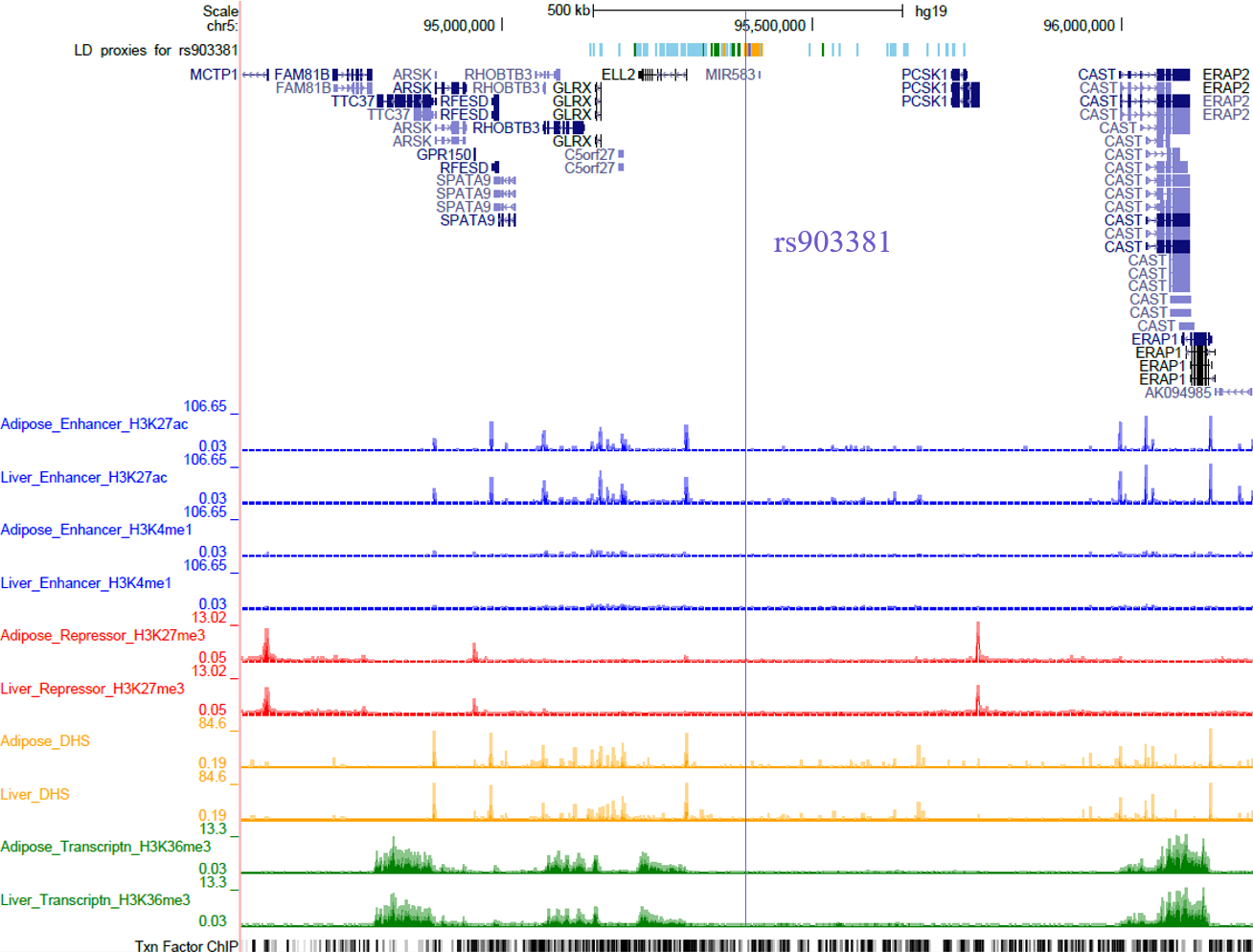


(I)


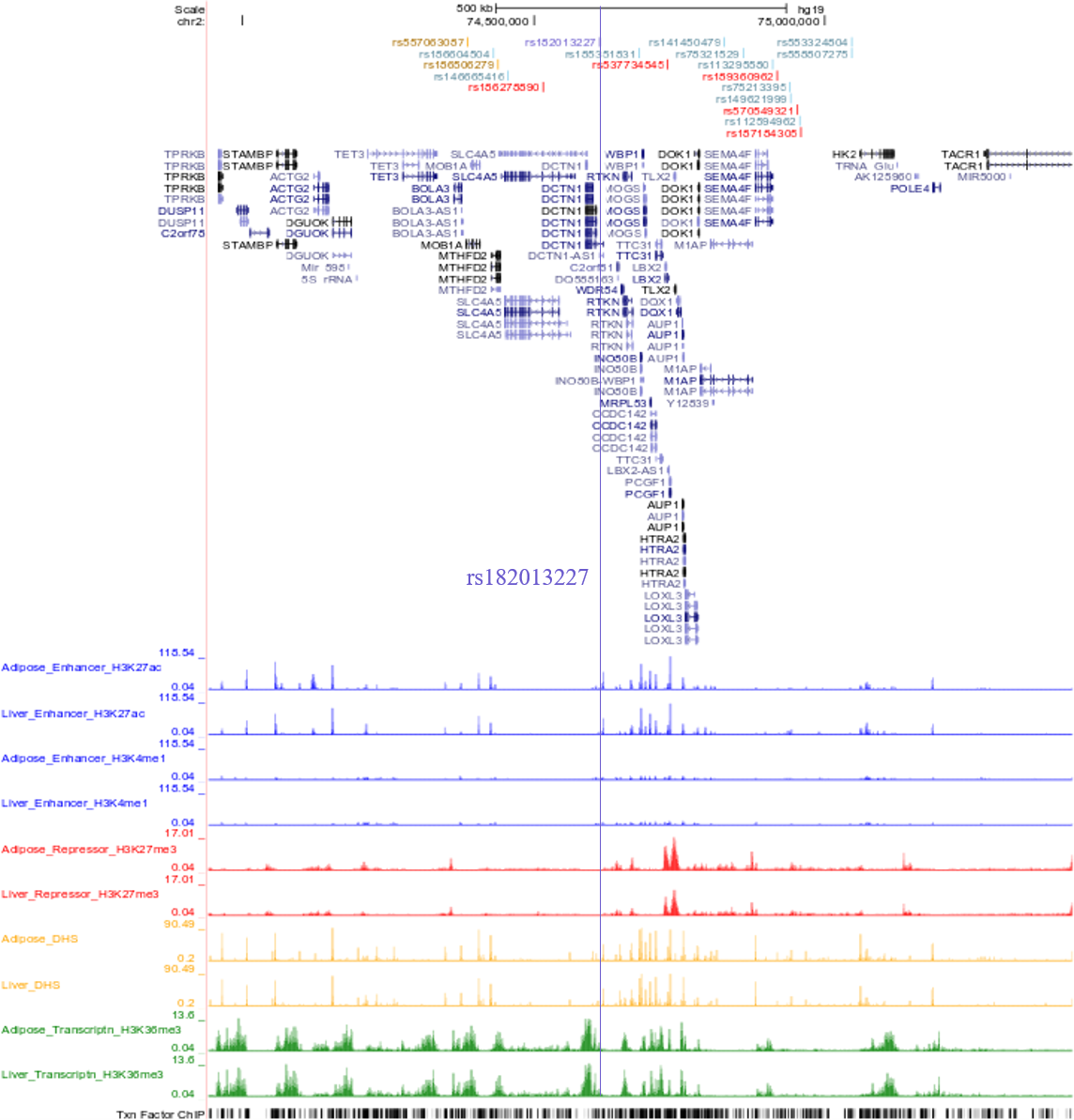

Supplement: S3 Fig — The top hit at each locus was colored purple, and the SNPs showing r2≥0.8, 0.8>r2≥0.6, 0.6>r2≥0.4 and 0.4>r2≥0.2 were colored red, orange, green and blue, respectively. (A) 5q31; (B) DLC1; (C) ZCCHC6; (D) DDHD1; (E) HLF; (F) B4GALNT3; (G) GPCPD1; (H) PCSK1; (I) MTHFD2. (DOCX) [file pgen.1008684.s003.docx]
