# Supplementary material for: Minority-centric meta-analyses of blood lipid levels identify novel loci in the Population Architecture using Genomics and Epidemiology (PAGE) study
Source: PLoS Genet. 2020 Mar 30;16(3):e1008684. doi: 10.1371/journal.pgen.1008684 (PMC7145272; doi:10.1371/journal.pgen.1008684)
Supplement: S1 Text — (DOCX) [file pgen.1008684.s021.docx]

**Supplementary Methods**

**Study descriptions**

1. The Atherosclerosis Risk in Communities Study (ARIC)

ARIC is a prospective epidemiologic study conducted in four U.S. communities[1]. It is designed to investigate the causes of atherosclerosis and its clinical outcomes, and variation in cardiovascular risk factors, medical care, and disease by race, gender, location, and date. ARIC includes two parts: the Cohort Component and the Community Surveillance Component. The Cohort Component began in 1987, and each ARIC field center randomly selected and recruited a cohort sample of approximately 4,000 individuals aged 45-64 from a defined population in their community, to receive extensive examinations, including medical, social, and demographic data. Follow-up currently occurs semi-annually, by telephone, to maintain contact and to assess health status of the cohort.

2. The BioME^TM^ Biobank (BioMe)

The Charles Bronfman Institute for Personalized Medicine at Mount Sinai Medical Center (MSMC), BioMe^TM^ BioBank (BioMe) is an EMR-linked bio-repository drawing from Mount Sinai Medical Center consented patients which were drawn from a population of over 70,000 inpatients and 800,000 outpatients annually (https://icahn.mssm.edu/research/ipm/programs/biome-biobank). The MSMC serves diverse local communities of upper Manhattan, including Central Harlem (86% African American), East Harlem (88% Hispanic/Latino), and Upper East Side (88% Caucasian/White) with broad health disparities. BioMe^TM^ enrolled over 26,500 participants from September 2007 through August 2013, with 25% African American, 36% Hispanic/Latino (primarily of Caribbean origin), 30% Caucasian, and 9% of other ancestry. The BioMe^TM^ population reflects community-level disease burdens and health disparities with broad public health impact. Biobank operations are fully integrated in clinical care processes, including direct recruitment from clinical sites waiting areas and phlebotomy stations by dedicate Biobank recruiters independent of clinical care providers, prior to or following a clinician standard of care visit. Recruitment currently occurs at a broad spectrum of over 30 clinical care sites.

3. The Coronary Artery Risk Development in Young Adults Study (CARDIA)

CARDIA examines the development and determinants of clinical and subclinical cardiovascular disease and its risk factors[2]. It began in 1985-6 with a group of 5,115 black and white men and women aged 18-30 years. The participants were selected so that there would be approximately the same number of people in subgroups of race, gender, education (high school or less and more than high school) and age (18-24 and 25-30) in each of 4 centers: Birmingham, AL; Chicago, IL; Minneapolis, MN; and Oakland, CA. These same participants were asked to participate in follow-up examinations during 1987-1988 (Year 2), 1990-1991 (Year 5), 1992-1993 (Year 7), 1995-1996 (Year 10), 2000-2001 (Year 15), and 2005-2006 (Year 20). A majority of the group has been examined at each of the follow-up examinations (91%, 86%, 81%, 79%, 74%, and 72%, respectively). Data have been collected on a variety of factors believed to be related to heart disease, such as blood pressure, cholesterol and other lipids, and glucose. Data have also been collected on physical measurements such as weight and skinfold fat as well as lifestyle factors such as substance use (tobacco and alcohol), dietary and exercise patterns, behavioral and psychological variables, medical and family history, and other chemistries (e.g., insulin).

4. The Hispanic Community Health Study/Study of Latinos (HCHS/SOL)

HCHS/SOL is a multi-center study of Hispanic/Latino populations with the goal of determining the role of acculturation in the prevalence and development of diseases, and to identify other traits that impact Hispanic/Latino health[3]. The study is sponsored by the National Heart, Lung, and Blood Institute (NHLBI) and other institutes, centers, and offices of the National Institutes of Health (NIH). Recruitment began in 2006 with a target population of 16,000 persons of Cuban, Puerto Rican, Dominican, Mexican or Central/South American origin. Household sampling was employed as part of the study design. Participants were recruited through four sites affiliated with San Diego State University, Northwestern University in Chicago, Albert Einstein College of Medicine in Bronx, New York, and the University of Miami. Researchers from seven academic centers provided scientific and logistical support. Study participants who were self-identified Hispanic/Latino and aged 18-74 years underwent extensive psycho-social and clinical assessments during 2008-2011. A re-examination of the HCHS/SOL cohort is conducted during 2015-2017. Annual telephone follow-up interviews are ongoing since study inception to determine health outcomes of interest.

5. The Multiethnic Cohort (MEC)

MEC is a population-based prospective cohort study including approximately 215,000 men and women from Hawaii and California[4]. All participants were 45-75 years of age at baseline, and primarily of 5 ancestries: Japanese Americans, African Americans, European Americans, Hispanic/Latinos, and Native Hawaiians. MEC was funded by the National Cancer Institute in 1993 to examine lifestyle risk factors and genetic susceptibility to cancer. All eligible cohort members completed baseline and follow-up questionnaires. Participants from the MEC sample that were genotyped on the MEGA chip and had lipid phenotypes included, 2,111 Africans, 2,122 Hispanic/Latinos, 283 Asians, 24 Native Americans, and 360 of other ancestry. An additional 482 Africans, and 3,455 Hispanics from MEC were genotyped on the Illumina Human1M-Duo chip and also have lipid phenotypes available. All genotyped samples were imputed to 1000 genomes phase 3.

6. The Women’s Health Initiative (WHI)

WHI is a long-term, prospective, multi-center cohort study investigating post-menopausal women’s health in the US[5]. WHI was funded by the National Institutes of Health and the National Heart, Lung, and Blood Institute to study strategies to prevent heart disease, breast cancer, colon cancer, and osteoporotic fractures in women 50-79 years of age. WHI involves 161,808 women recruited between 1993 and 1998 at 40 centers across the US. The study consists of two parts: the WHI Clinical Trial which was a randomized clinical trial of hormone therapy, dietary modification, and calcium/Vitamin D supplementation, and the WHI Observational Study, which focused on many of the inequities in women’s health research and provided practical information about incidence, risk factors, and interventions related to heart disease, cancer, and osteoporotic fractures.

There were five WHI ancillary studies included in the meta-analysis, namely the Genomics and Randomized Trials Network (GARNET), the Hip Fracture GWAS (HIPFX), the Long Life Study (LLS), the Women's Health Initiative Memory Study (WHIMS) and the Women's Health Initiative - SNP Health Association Resource (WHI-SHARe). The GARNET study is series of genome-wide association studies of treatment response in randomized clinical trials, aiming to identify genetic variants associated with response to treatments for conditions of clinical or public health significance. The HIPFX study was designed to perform epidemiological studies of hip fracture in women. The LLS study included 7,875 women from the WHI Extension II Medical Records Cohort (MRC). The LLS consisted of a one-time in-person visit (conducted sometime between March 2012 and May 2013) with a blood draw, a brief clinical assessment, and an assessment of functional status. The WHIMS study is a trial to examine the effect of estrogen therapy in preventing and slowing the progression of dementia. The WHI-SHARe study is part of NHLBI's SNP Health Association Resource (SHARe) project, aiming to enhance the statistical power for research specific to groups defined by race and ethnicity and to discover or replicate genes associated with quantitative traits (such as blood pressure and blood lipids) in these groups. The participants in GARNET, HIPFX, LLS and WHIMS are of European descent while the ones in WHI-SHARe are of African descent.

7. The Million Veteran Program

The Million Veteran Program is a nation-wide research program established by the Veteran Affairs Office of Research and Development in 2011[6]. The GWAS analyses for the four lipid traits in MVP were previously published[7]. The list of MVP Consortium was provided below.

Million Veteran Program Consortium

*MVP Executive Committee*

-Co-Chair: J. Michael Gaziano, M.D., M.P.H.

-Co-Chair: Rachel Ramoni, D.M.D., Sc.D.

-Jim Breeling, M.D. (ex-officio)

-Kyong-Mi Chang, M.D.

-Grant Huang, Ph.D.

-Sumitra Muralidhar, Ph.D.

-Christopher J. O’Donnell, M.D., M.P.H.

-Philip S. Tsao, Ph.D.

*MVP Program Office*

-Sumitra Muralidhar, Ph.D.

-Jennifer Moser, Ph.D.

*MVP Recruitment/Enrollment*

-Recruitment/Enrollment Director/Deputy Director, Boston – Stacey B.

Whitbourne, Ph.D.; Jessica V. Brewer, M.P.H.

-MVP Coordinating Centers

Clinical Epidemiology Research Center (CERC), West Haven – John Concato, M.D., M.P.H.

Cooperative Studies Program Clinical Research Pharmacy Coordinating Center, Albuquerque - Stuart Warren, J.D., Pharm D.; Dean P. Argyres, M.S.

Genomics Coordinating Center, Palo Alto – Philip S. Tsao, Ph.D.

Massachusetts Veterans Epidemiology Research Information Center (MAVERIC), Boston - J. Michael Gaziano, M.D., M.P.H.

MVP Information Center, Canandaigua – Brady Stephens, M.S.

-Core Biorepository, Boston – Mary T. Brophy M.D., M.P.H.; Donald E. Humphries, Ph.D.

-MVP Informatics, Boston – Nhan Do, M.D.; Shahpoor Shayan

-Data Operations/Analytics, Boston – Xuan-Mai T. Nguyen, Ph.D.

*MVP Science*

-Genomics - Christopher J. O’Donnell, M.D., M.P.H.; Saiju Pyarajan Ph.D.; Philip S. Tsao, Ph.D.

-Phenomics - Kelly Cho, M.P.H, Ph.D.

-Data and Computational Sciences – Saiju Pyarajan, Ph.D.

-Statistical Genetics – Elizabeth Hauser, Ph.D.; Yan Sun, Ph.D.; Hongyu Zhao, Ph.D.

*MVP Local Site Investigators*

-Atlanta VA Medical Center (Peter Wilson)

1670 Clairmont Rd, Decatur, GA 30033

-Bay Pines VA Healthcare System(Rachel McArdle)

10,000 Bay Pines Blvd Bay Pines FL 33744

-Birmingham VA Medical Center (Louis Dellitalia)

700 S. 19th Street Birmingham AL 35233

-Cincinnati VA Medical Center (John Harley)

3200 Vine Street, Cincinnati, OH 45220

-Clement J. Zablocki VA Medical Center (Jeffrey Whittle)

5000 West National Avenue, Milwaukee, WI 53295

-Durham VA Medical Center (Jean Beckham)

508 Fulton Street Durham, NC 27705

-Edith Nourse Rogers Memorial Veterans Hospital (John Wells)

200 Springs Road, Bedford, MA 01730

-Edward Hines, Jr. VA Medical Center (Salvador Gutierrez)

5000 South 5th Avenue, Hines, IL 60141

-Fayetteville VA Medical Center (Gretchen Gibson)

1100 N College Ave, Fayetteville, AR 72703

-VA Health Care Upstate New York (Laurence Kaminsky)

113 Holland Avenue Albany NY 12208

-New Mexico VA Health Care System (Gerardo Villareal)

1501 San Pedro Drive, S.E.Albuquerque, NM 87108

-VA Boston Healthcare System (Scott Kinlay)

150 S. Huntington Avenue, Boston, MA 02130

-VA Western New York Healthcare System (Junzhe Xu)

3495 Bailey Avenue Buffalo, NY 14215-1199

-Ralph H. Johnson VA Medical Center (Mark Hamner)

109 Bee Street, Mental Health Research, Charleston, SC 29401

-Wm. Jennings Bryan Dorn VA Medical Center (Kathlyn Sue Haddock)

6439 Garners Ferry Road, Columbia, SC 29209

-VA North Texas Health Care System (Sujata Bhushan)

4500 S. LANCASTER ROAD, DALLAS, TX 75216

-Hampton VA Medical Center (Pran Iruvanti)

100 Emancipation Drive, Hampton, VA 23667

-Hunter Holmes McGuire VA Medical Center (Michael Godschalk)

1201 Broad Rock Blvd., Richmond, VA 23249

-Iowa City VA Health Care System (Zuhair Ballas)

601 Highway 6 West, Iowa City, IA 52246-2208

-Jack C. Montgomery VA Medical Center (Malcolm Buford)

1011 Honor Heights Dr., Muskogee, OK 74401

-James A. Haley Veterans’ Hospital (Stephen Mastorides)

13000 Bruce B. Downs Blvd., Tampa, FL 33612

-Louisville VA Medical Center (Jon Klein)

800 Zorn Avenue, Louisville, KY 40206

-Manchester VA Medical Center (Nora Ratcliffe)

718 Smyth Road, Manchester, NH 03104

-Miami VA Health Care System (Hermes Florez)

1201 NW 16th Street, 11 GRC, Miami FL 33125

-Michael E. DeBakey VA Medical Center (Alan Swann)

2002 Holcombe Blvd. Houston TX 77030

-Minneapolis VA Health Care System (Maureen Murdoch)

One Veterans Drive Minneapolis MN 55417

-N. FL/S. GA Veterans Health System (Peruvemba Sriram)

1601 SW Archer Road, Gainesville, FL 32608

-Northport VA Medical Center (Shing Shing Yeh)

79 Middleville Road, Northport, NY 11768

-Overton Brooks VA Medical Center (Ronald Washburn)

510 East Stoner Ave, Shreveport, LA 71101

-Philadelphia VA Medical Center (Darshana Jhala)

3900 Woodland Avenue, Philadelphia, PA 19104

-Phoenix VA Health Care System (Samuel Aguayo)

650 E. Indian School Road, Phoenix, AZ 85012

-Portland VA Medical Center (David Cohen)

3710 SW U.S. Veterans Hospital Road, Portland, OR 97239

-Providence VA Medical Center (Satish Sharma)

830 Chalkstone Avenue, Providence, RI 02908

-Richard Roudebush VA Medical Center (John Callaghan)

1481 West 10th Street, Indianapolis, IN 46202

-Salem VA Medical Center (Kris Ann Oursler)

1970 Roanoke Blvd.,Salem, VA 24153

-San Francisco VA Health Care System (Mary Whooley)

4150 Clement Street, San Francisco, CA 94121

-South Texas Veterans Health Care System (Sunil Ahuja)

7400 Merton Minter Boulevard, San Antonio, TX 78229

-Southeast Louisiana Veterans Health Care System (Amparo Gutierrez)

2400 Canal Street, New Orleans, LA 70119

-Southern Arizona VA Health Care System (Ronald Schifman)

3601 S 6th Ave, Tucson, AZ 85723

-Sioux Falls VA Health Care System (Jennifer Greco)

2501 W 22nd St, Sioux Falls, SD 57105

-St. Louis VA Health Care System (Michael Rauchman)

915 North Grand Blvd., St. Louis, MO 63106

-Syracuse VA Medical Center (Richard Servatius)

800 Irving Avenue, Syracuse, NY 13210

-VA Eastern Kansas Health Care System (Mary Oehlert)

4101 S 4th Street Trafficway, Leavenworth, KS 66048

-VA Greater Los Angeles Health Care System (Agnes Wallbom)

11301 Wilshire Blvd Los Angeles, CA 90073

-VA Loma Linda Healthcare System (Ronald Fernando)

11201 Benton Street, Loma Linda, CA 92357

-VA Long Beach Healthcare System (Timothy Morgan)

5901 East 7th Street Long Beach CA 90822

-VA Maine Healthcare System (Todd Stapley)

1 VA Center, Augusta, ME 04330

-VA New York Harbor Healthcare System (Scott Sherman)

423 East 23rd Street New York, NY 10010

-VA Pacific Islands Health Care System (Gwenevere Anderson)

459 Patterson Rd, Honolulu, HI 96819

-VA Palo Alto Health Care System (Philip Tsao)

3801 Miranda Avenue Palo Alto, CA 94304-1290

-VA Pittsburgh Health Care System (Elif Sonel)

University Drive, Pittsburgh, PA 15240

-VA Puget Sound Health Care System (Edward Boyko)

1660 S. Columbian Way Seattle, WA 98108-1597

-VA Salt Lake City Health Care System (Laurence Meyer)

500 Foothill Drive Salt Lake City, UT 84148

-VA San Diego Healthcare System (Samir Gupta)

3350 La Jolla Village Drive, San Diego, CA 92161

-VA Southern Nevada Healthcare System (Joseph Fayad)

6900 North Pecos Road, North Las Vegas, NV 89086

-VA Tennessee Valley Healthcare System (Adriana Hung)

1310 24th Ave. South Nashville, TN 37212

-Washington DC VA Medical Center (Jack Lichy)

50 Irving St, Washington, D. C. 20422

-W.G. (Bill) Hefner VA Medical Center (Robin Hurley)

1601 Brenner Ave, Salisbury, NC 28144

-White River Junction VA Medical Center (Brooks Robey)

163 Veterans Drive, White River Junction, VT 05009

-William S. Middleton Memorial Veterans Hospital (Robert Striker)

2500 Overlook Terrace, Madison, WI 53705

8. The Global Hispanic Lipids Consortium

The Global Hispanic Lipids Consortium included over 10,000 Hispanic ancestry participants from 11 studies and was used for replication of the PAGE novel findings. The included studies are Genetics of Latinos Diabetic Retinopathy (GOLDR, N=597)[8], Mexican-American Hypertension-Insulin Resistance Family Study (HTN-IR, N=732)[9-11], Insulin Resistance Atherosclerosis Study Family Study (IRASFS, N=1,019)[12], Insulin Resistance Atherosclerosis Study (IRASc, N=176)[13], Los Angeles Latino Eye Study (LALES, N=1,484)[14], Mexican-American Coronary Artery Disease (MACAD, N=730)[15, 16], Multi-Ethnic Study of Atherosclerosis (MESA, N=1,422)[17, 18], Mexico City sample 1 (MC1, N=1,278)[19], Mexico City sample 2 (MC2, N=1,783)[19], Non-Insulin-Dependent Diabetes Mellitus-Atherosclerosis (NIDDM, N=241)[16] and Starr County Health Studies (SC, N=544)[19]. Transformation of lipid levels were detailed previously[20]. Association analyses were performed in each individual study and the results were then combined in a mega-regression using MR-MEGA[21], including one covariate to capture study-level differences in ancestry.

9. Electronic Medical Records and Genomics (eMERGE) Network

A total of 83,717 individuals are present in the eMERGE Phase III EHR-linked imputed genotype dataset[22]. We have restricted the analyses to adult participants (>18 years of age) of three mutually exclusive ethnic groups, namely, non-Hispanic European Americans, non-Hispanic African Americans (matching PCA-based k-means group and self-reported ancestry, self-identified to be “non-Hispanic”), and Hispanic Americans (self-identified as “Hispanic”). eMERGE participants from Mount Sinai BioME dataset overlapping with PAGE Consortium dataset were excluded. Blood lipid measurements, low-density lipoprotein cholesterol (LDL-C), high-density lipoprotein cholesterol (HDL-C), total cholesterol (TC), and triglycerides (TG) were extracted from the electronic health record (EHR) and participants without any lipid data in the EHR were excluded. Lipid values ≤ 0 mg/dl and extreme outliers (LDL-C>380 mg/dl, HDL-C>120 mg/dl, TC> 480 mg/dl, TG > 1000 mg/dl) were removed from the dataset. For each participant, LDL-C values with a concurrent TG value > 400 mg/dl were also removed due to inaccuracy of Freidewald equation in presence of elevated serum triglyceride levels. For individuals with multiple measurements, maximum LDL-C, TC, TG values and minimum HDL-C value were selected for the analyses in order to ascertain pre-treatment lipid values and reduce confounding from lipid-lowering therapy. In case of statin use documented in the EHR within 90 days prior to the measurement date of LDL-C and TC, values were adjusted for lipid-lowering therapy by dividing by 0.7 and 0.8, respectively. TG levels were transformed by natural log, while TC, HDL-C, and LDL-C LEVELS were transformed by the inverse normal distribution. The association of the quantitative traits with selected SNPs was conducted within each ancestral group (Hispanic, European, and African) by regressing each of the transformed traits on the covariates age (10-year groupings), sex, and 10 principal components. The residuals from these regressions were then used as adjusted traits for testing their association with SNPs. Because kinship coefficients suggested that some subjects were genetically related, we accounted for relationships by using the estimated identity by descent coefficients to correct the relationships[23]. All imputed SNPs were processed in our Mayo QC pipeline, with processing conducted separately for each of the ancestry groups[24]. SNPs with R^2^ ≥0.3 were included in the replication analysis.

10. Jackson Heart Study (JHS)

JHS is a longitudinal, population-based cohort, which was designed for prospective research into the epidemiology and determinants of cardiovascular disease (CVD) in African American (AA) populations from the Jackson, Mississippi metropolitan area[25]. The design and sampling of JHS included participants from the Jackson cohort of the U.S. Atherosclerosis Risk in Communities Study (ARIC), a random component, a family component, and a structured volunteer sample[26]. Study baseline data collection began in late 2000 and was completed in early 2004, and a total of 5,306 male and female participants were recruited. The baseline examination included a home interview, a clinic visit, laboratory tests, complete blood cell counts and a physical examination. Outliers based on principal components (PCs), sex inconsistencies, duplicates, sample swaps based on discordance with known pedigrees, and one of each pair of monozygotic twins were excluded from genetic analyses. Genotyped samples were imputed to 1000 Genome Phase 3v5 reference panel using the Michigan Imputation Server (https://imputationserver.sph.umich.edu/index.html#!pages/home). SNPs with R^2^ ≥0.3 were included in the replication analysis. Adjustment for medication and trait transformation were performed according to recommendations from the Global Lipids Genetics Consortium (GLGC). In brief, TG levels were natural log transformed, and TC and LDL levels were adjusted for statin use (TC_adjust = TC_raw/0.8, LDL_adjust = TC_adjust -HDL – (TG/5), LDL levels were set to missing if TG ≥400mg/dL). Association analyses of the nine novel loci with inverse normalized lipid levels were performed in EPACTS v3.2.6 using the EMMAX test (http://csg.sph.umich.edu/kang/epacts/download/), with adjustment for age, sex, and the first four PCs.

11. Kaiser Permanente Research Bank

Summary statistics of the Kaiser study[27] were downloaded from the GWAS Catalog website (https://www.ebi.ac.uk/gwas/publications/29507422). Only ancestry-combined results were available.

12. UK BioBank (UKBB)

There were three ancestral groups that were included in our replication analysis, namely European, African and Asian. Summary statistics in European populations are available at <http://www.nealelab.is/uk-biobank>. For African and Asian ancestry populations, the version 2 imputed data was used (https://biobank.ctsu.ox.ac.uk/crystal/label.cgi?id=100319). Participants were grouped as Africans or Asians based on their ancestry coding [African: coding=4 (Black or Black British), 2001 (White and Black Caribbean), 2002 (White and Black African), 4001 (Caribbean), 4002 (African) or 4003 (Any other Black background); Asian: coding=3 (Asian or Asian British), 5 (Chinese), 2003 (White and Asian), 3001 (Indian), 3002 (Pakistani), 3003 (Bangladeshi) or 3004 (Any other Asian background)]. Participants were divided into four subgroups based on lipid-lowering medication use and gender in African and Asian groups, respectively. Raw lipid levels were winsorized (cutoff: mean±3SD) and inverse normalized residuals were estimated with adjustment for age in each subgroup. Association analyses of the nine novel loci with the residuals were performed with adjustment of 10 PCs in each subgroup. Summary statistics from the four subgroups were then combined through inverse-variance-weighted meta-analysis in African and Asian groups, respectively.

**Bioinformatic functional follow-up**

Bioinformatic functional follow-up was performed for each novel locus using our comprehensive functional annotation database and a custom UCSC analysis data hub. Our functional database was constructed using WGSA[28] to annotate variants with various functional annotations pertaining to gene-centric function (GTEx[29] and SPIDEX [30]) and genome-wide functional prediction scores (DANN[31] and EigenPC[32]). Variants with DANN rank score ≥ 0.9 were coded as deleterious, and variants with Eigen PC phred score ≥ 17 were coded as functional. Custom UCSC bed tracks included the top hit of each novel locus and the proxy variants that were in LD (r^2^≥0.2) with the top hit within ±1Mb region. The LD proxies of the 9 novel loci were generated using either ethnic-specific or ethnic-combined data (pooled MEGA minority data used for *5q31*, AA-specific data used for *ZCCHC6* and *MTHFD2*, Hispanic-specific data used for *DLC1* and *PCSK1*, Hawaiian-specific data used for *DDHD1*, and weighted MEGA minority and European data used for *HLF*, *B4GALNT3* and *GPCPD1*). Adult liver and adipose tissue, the two most relevant tissues to lipid metabolism, were selected to examine chromatin immunoprecipitation-sequencing (ChiP-seq) signals associated with enhancers (H3K27ac and H3K4m1), repressors (H3K27me3), and transcribed regions (H3K36me3). In addition, the Txn Factor ChIP Track was used to visualize ChIP-seq transcription factor binding sites from the Encyclopedia of DNA Elements (ENCODE) collection, together with DNA binding motifs identified within these regions by the ENCODE Factorbook repository[33, 34].

**PrediXcan analysis**

In the PrediXcan analysis, we focused on adipose tissue (385 and 313 samples for subcutaneous and visceral, respectively), liver (153 samples) and whole blood (369 samples), which are closed linked to lipid metabolism. First, GREx in over 50,000 PAGE minority ancestry participants genotyped by MEGA were imputed using publicly available GTEx version 7-derived predictor models by PrediXcan software[35]. Elastic net regression was used to build the models, including all cis-SNPs (defined as within 1Mb of the gene) with MAF>5%, and only genes with significant prediction accuracy (FDR<0.05) were included in the models. There were 8,271, 6,594, 3,355 and 6,298 genes included in the models in subcutaneous adipose tissue, visceral adipose tissue, liver and whole blood, respectively. Associations between the GREx and the four lipid traits were then estimated using SUGEN both in ancestry-combined and ancestry-stratified manners. Genes with *P*<2.04E-6 [0.05/(8271+6594+3355+6298)] in the ancestry-combined analysis were considered as significant.

**References**

1. Williams OD. The Atherosclerosis Risk in Communities (Aric) Study - Design and Objectives. American Journal of Epidemiology. 1989;129(4):687-702. PubMed PMID: WOS:A1989T805200005.

2. Friedman GD, Cutter GR, Donahue RP, Hughes GH, Hulley SB, Jacobs DR, et al. Cardia - Study Design, Recruitment, and Some Characteristics of the Examined Subjects. J Clin Epidemiol. 1988;41(11):1105-16. doi: Doi 10.1016/0895-4356(88)90080-7. PubMed PMID: WOS:A1988R520300009.

3. Lavange LM, Kalsbeek WD, Sorlie PD, Aviles-Santa LM, Kaplan RC, Barnhart J, et al. Sample Design and Cohort Selection in the Hispanic Community Health Study/Study of Latinos. Ann Epidemiol. 2010;20(8):642-9. doi: 10.1016/j.annepidem.2010.05.006. PubMed PMID: WOS:000280131600009.

4. Gram IT, Park SY, Kolonel LN, Maskarinec G, Wilkens LR, Henderson BE, et al. Smoking and Risk of Breast Cancer in a Racially/Ethnically Diverse Population of Mainly Women Who Do Not Drink Alcohol. American Journal of Epidemiology. 2015;182(11):917-25. doi: 10.1093/aje/kwv092. PubMed PMID: WOS:000366371700005.

5. Anderson G, Cummings S, Freedman LS, Furberg C, Henderson M, Johnson SR, et al. Design of the Women's Health Initiative Clinical Trial and Observational Study. Controlled Clinical Trials. 1998;19(1):61-109. PubMed PMID: WOS:000071850900006.

6. Gaziano JM, Concato J, Brophy M, Fiore L, Pyarajan S, Breeling J, et al. Million Veteran Program: A mega-biobank to study genetic influences on health and disease. J Clin Epidemiol. 2016;70:214-23. Epub 2015/10/07. doi: 10.1016/j.jclinepi.2015.09.016. PubMed PMID: 26441289.

7. Klarin D, Damrauer SM, Cho K, Sun YV, Teslovich TM, Honerlaw J, et al. Genetics of blood lipids among ~300,000 multi-ethnic participants of the Million Veteran Program. Nat Genet. 2018. Epub 2018/10/03. doi: 10.1038/s41588-018-0222-9. PubMed PMID: 30275531.

8. Kuo JZ, Guo X, Klein R, Klein BE, Cui J, Rotter JI, et al. Systemic soluble tumor necrosis factor receptors 1 and 2 are associated with severity of diabetic retinopathy in Hispanics. Ophthalmology. 2012;119(5):1041-6. Epub 2012/02/15. doi: 10.1016/j.ophtha.2011.10.040. PubMed PMID: 22330960; PubMed Central PMCID: PMCPMC3343221.

9. Xiang AH, Azen SP, Raffel LJ, Tan S, Cheng LS, Diaz J, et al. Evidence for joint genetic control of insulin sensitivity and systolic blood pressure in hispanic families with a hypertensive proband. Circulation. 2001;103(1):78-83. Epub 2001/01/04. PubMed PMID: 11136689.

10. Cheng LS, Davis RC, Raffel LJ, Xiang AH, Wang N, Quinones M, et al. Coincident linkage of fasting plasma insulin and blood pressure to chromosome 7q in hypertensive hispanic families. Circulation. 2001;104(11):1255-60. Epub 2001/09/12. PubMed PMID: 11551876.

11. Pojoga LH, Underwood PC, Goodarzi MO, Williams JS, Adler GK, Jeunemaitre X, et al. Variants of the caveolin-1 gene: a translational investigation linking insulin resistance and hypertension. J Clin Endocrinol Metab. 2011;96(8):E1288-92. Epub 2011/05/27. doi: 10.1210/jc.2010-2738. PubMed PMID: 21613355; PubMed Central PMCID: PMCPMC3146791.

12. Henkin L, Bergman RN, Bowden DW, Ellsworth DL, Haffner SM, Langefeld CD, et al. Genetic epidemiology of insulin resistance and visceral adiposity. The IRAS Family Study design and methods. Ann Epidemiol. 2003;13(4):211-7. Epub 2003/04/10. PubMed PMID: 12684185.

13. Wagenknecht LE, Mayer EJ, Rewers M, Haffner S, Selby J, Borok GM, et al. The insulin resistance atherosclerosis study (IRAS) objectives, design, and recruitment results. Ann Epidemiol. 1995;5(6):464-72. Epub 1995/11/01. PubMed PMID: 8680609.

14. Varma R, Torres M, Pena F, Klein R, Azen SP, Los Angeles Latino Eye Study G. Prevalence of diabetic retinopathy in adult Latinos: the Los Angeles Latino eye study. Ophthalmology. 2004;111(7):1298-306. Epub 2004/07/06. doi: 10.1016/j.ophtha.2004.03.002. PubMed PMID: 15234129.

15. Goodarzi MO, Guo XQ, Taylor KD, Quinones MJ, Samayoa C, Yang HY, et al. Determination and use of haplotypes: Ethnic comparison and association of the lipoprotein lipase gene and coronary artery disease in Mexican-Americans. Genetics in Medicine. 2003;5(4):322-7. doi: 10.1097/01.Gim.0000076971.55421.Ad. PubMed PMID: WOS:000184636500008.

16. Palmer ND, Goodarzi MO, Langefeld CD, Wang N, Guo X, Taylor KD, et al. Genetic Variants Associated With Quantitative Glucose Homeostasis Traits Translate to Type 2 Diabetes in Mexican Americans: The GUARDIAN (Genetics Underlying Diabetes in Hispanics) Consortium. Diabetes. 2015;64(5):1853-66. Epub 2014/12/20. doi: 10.2337/db14-0732. PubMed PMID: 25524916; PubMed Central PMCID: PMCPMC4407862.

17. Bild DE, Bluemke DA, Burke GL, Detrano R, Diez Roux AV, Folsom AR, et al. Multi-Ethnic Study of Atherosclerosis: objectives and design. Am J Epidemiol. 2002;156(9):871-81. Epub 2002/10/25. PubMed PMID: 12397006.

18. Wang Z, Manichukal A, Goff DC, Jr., Mora S, Ordovas JM, Pajewski NM, et al. Genetic associations with lipoprotein subfraction measures differ by ethnicity in the multi-ethnic study of atherosclerosis (MESA). Hum Genet. 2017;136(6):715-26. Epub 2017/03/30. doi: 10.1007/s00439-017-1782-y. PubMed PMID: 28352986; PubMed Central PMCID: PMCPMC5429342.

19. Below JE, Parra EJ, Gamazon ER, Torres J, Krithika S, Candille S, et al. Meta-analysis of lipid-traits in Hispanics identifies novel loci, population-specific effects, and tissue-specific enrichment of eQTLs. Sci Rep. 2016;6:19429. Epub 2016/01/20. doi: 10.1038/srep19429. PubMed PMID: 26780889; PubMed Central PMCID: PMCPMC4726092.

20. Below JE, Parra EJ, Gamazon ER, Torres J, Krithika S, Candille S, et al. Meta-analysis of lipid-traits in Hispanics identifies novel loci, population-specific effects, and tissue-specific enrichment of eQTLs. Sci Rep-Uk. 2016;6. doi: 10.1038/srep19429. PubMed PMID: WOS:000368335800001.

21. Magi R, Horikoshi M, Sofer T, Mahajan A, Kitajima H, Franceschini N, et al. Trans-ethnic meta-regression of genome-wide association studies accounting for ancestry increases power for discovery and improves fine-mapping resolution. Hum Mol Genet. 2017;26(18):3639-50. Epub 2017/09/16. doi: 10.1093/hmg/ddx280. PubMed PMID: 28911207; PubMed Central PMCID: PMCPMC5755684.

22. Stanaway IB, Hall TO, Rosenthal EA, Palmer M, Naranbhai V, Knevel R, et al. The eMERGE genotype set of 83,717 subjects imputed to ~40 million variants genome wide and association with the herpes zoster medical record phenotype. Genet Epidemiol. 2019;43(1):63-81. Epub 2018/10/10. doi: 10.1002/gepi.22167. PubMed PMID: 30298529.

23. Schaid DJ, McDonnell SK, Sinnwell JP, Thibodeau SN. Multiple Genetic Variant Association Testing by Collapsing and Kernel Methods With Pedigree or Population Structured Data. Genetic Epidemiology. 2013;37(5):409-18. doi: 10.1002/gepi.21727. PubMed PMID: WOS:000320386800001.

24. Stanaway IB, Hall TO, Rosenthal EA, Palmer M, Naranbhai V, Knevel R, et al. The eMERGE genotype set of 83,717 subjects imputed to similar to 40 million variants genome wide and association with the herpes zoster medical record phenotype. Genetic Epidemiology. 2019;43(1):63-81. doi: 10.1002/gepi.22167. PubMed PMID: WOS:000455511900005.

25. Taylor HA, Jr., Wilson JG, Jones DW, Sarpong DF, Srinivasan A, Garrison RJ, et al. Toward resolution of cardiovascular health disparities in African Americans: design and methods of the Jackson Heart Study. Ethn Dis. 2005;15(4 Suppl 6):S6-4-17. Epub 2005/12/02. PubMed PMID: 16320381.

26. Sempos CT, Bild DE, Manolio TA. Overview of the Jackson Heart Study: a study of cardiovascular diseases in African American men and women. Am J Med Sci. 1999;317(3):142-6. Epub 1999/04/01. doi: 10.1097/00000441-199903000-00002. PubMed PMID: 10100686.

27. Hoffmann TJ, Theusch E, Haldar T, Ranatunga DK, Jorgenson E, Medina MW, et al. A large electronic-health-record-based genome-wide study of serum lipids. Nature Genetics. 2018;50(3):401-+. doi: 10.1038/s41588-018-0064-5. PubMed PMID: WOS:000427933400016.

28. Liu X, White S, Peng B, Johnson AD, Brody JA, Li AH, et al. WGSA: an annotation pipeline for human genome sequencing studies. J Med Genet. 2016;53(2):111-2. Epub 2015/09/24. doi: 10.1136/jmedgenet-2015-103423. PubMed PMID: 26395054; PubMed Central PMCID: PMCPMC5124490.

29. Gamazon ER, Segre AV, van de Bunt M, Wen XQ, Xi HS, Hormozdiari F, et al. Using an atlas of gene regulation across 44 human tissues to inform complex disease- and trait-associated variation. Nature Genetics. 2018;50(7):956-+. doi: 10.1038/s41588-018-0154-4. PubMed PMID: WOS:000437224400011.

30. Xiong HY, Alipanahi B, Lee LJ, Bretschneider H, Merico D, Yuen RKC, et al. The human splicing code reveals new insights into the genetic determinants of disease. Science. 2015;347(6218). doi: ARTN 1254806

10.1126/science.1254806. PubMed PMID: WOS:000347918900035.

31. Quang D, Chen YF, Xie XH. DANN: a deep learning approach for annotating the pathogenicity of genetic variants. Bioinformatics. 2015;31(5):761-3. doi: 10.1093/bioinformatics/btu703. PubMed PMID: WOS:000352268500019.

32. Ionita-Laza I, McCallum K, Xu B, Buxbaum JD. A spectral approach integrating functional genomic annotations for coding and noncoding variants. Nature Genetics. 2016;48(2):214-20. doi: 10.1038/ng.3477. PubMed PMID: WOS:000369043900021.

33. Gerstein MB, Kundaje A, Hariharan M, Landt SG, Yan KK, Cheng C, et al. Architecture of the human regulatory network derived from ENCODE data. Nature. 2012;489(7414):91-100. doi: 10.1038/nature11245. PubMed PMID: WOS:000308347000042.

34. Wang J, Zhuang J, Iyer S, Lin X, Whitfield TW, Greven MC, et al. Sequence features and chromatin structure around the genomic regions bound by 119 human transcription factors. Genome Res. 2012;22(9):1798-812. Epub 2012/09/08. doi: 10.1101/gr.139105.112. PubMed PMID: 22955990; PubMed Central PMCID: PMCPMC3431495.

35. Gamazon ER, Wheeler HE, Shah KP, Mozaffari SV, Aquino-Michaels K, Carroll RJ, et al. A gene-based association method for mapping traits using reference transcriptome data. Nature Genetics. 2015;47(9):1091-+. doi: 10.1038/ng.3367. PubMed PMID: WOS:000360394100022.
