# Supplementary material for: Minority-centric meta-analyses of blood lipid levels identify novel loci in the Population Architecture using Genomics and Epidemiology (PAGE) study
Source: PLoS Genet. 2020 Mar 30;16(3):e1008684. doi: 10.1371/journal.pgen.1008684 (PMC7145272; doi:10.1371/journal.pgen.1008684)
Supplement: Attachment — Submitted filename: PAGE_lipids_rebuttal.docx [file pgen.1008684.s022.docx]

Reviewer #1: Hu et al. present results of GWAS analyses of lipid profiles in ethnic minorities in the PAGE Consortium. They identified novel loci, some of which are driven by one ancestry, and/or are rare or monomorphic in Europeans. Some of the novel loci were replicated in additional published ethnic-specific and trans-ethnic studies. Allelic effects on lipids were not heterogeneous across ethnicities. Loci were interrogated for potential causal genes and fine-mapped to localise potential causal variants. The study is very timely, and the focus on the genetics of lipids in minority groups is extremely important. The messages about fine-mapping and use of multi-ethnic risk scores are very important to the human genetics research community. However, I have some concerns over the analytical approaches used.

1. Trait transformations. The authors state that lipid levels were inverse-normally transformed by sex. It was not clear to me whether this meant inverse-rank normalisation, or a Z-score transformation. In either case, this means that the effect estimates obtained for each study are NOT on the same scale, so it was not clear to me that a fixed-effects meta-analysis with inverse-variance weighting of effect sizes was appropriate, and that a Stouffer fixed-effects meta-analysis would be more appropriate. There are also no details (that I could find) on trait transformations in the replication sets, but again the same concern remains about effect size differences between studies.

**Response: Thank you for your comments. We performed inverse-rank normalization to transform the lipid levels in the discovery analyses. In the replication stage, all participating studies used the same transformation strategy except for Kaiser. Details of trait transformation in each study were presented in the Supplementary Methods.**

**In order to implement the Stouffer fixed-effect approach, we performed sample-size-weighted meta-analysis using METAL. All nine novel loci remained genome-wide significant except for *B4GALNT3* and *GPCPD1*. However, these two novel loci were successfully confirmed in the replication stage. In addition, we are focusing on identifying novel loci associated with lipid traits instead of accurately estimating the effect sizes. As a result, we presented the inverse-variance-weighted meta-analysis results in Table 2 and the sample-size-weighted meta-analysis results in Supplementary Table 16. In the replication stage, we used sample-size-weighted meta-analyses since Kaiser used a different trait transformation strategy.**

2. Lack of replication. For several of the novel loci, there is very weak (if any) evidence of replication, even in ethnically matched components. I couldn’t find the sample sizes of the replication sets anywhere in the manuscript, but I’m assuming that they are substantially larger than PAGE? What is the explanation for this lack of replication – without this, I find it difficult to accept these associations as real.

**Response: We appreciate the reviewer’s comment and agree that successful replication of the novel findings is crucial. Three additional studies were included in the replication stage, namely the Jackson Heart Study (African ancestry), the Kaiser Permanente Research Bank (multi-ethnic) and the UK BioBank (European, African and Asian ancestry).** **After adding these three studies to our replication datasets, we confirmed three novel loci at genome-wide significance (*HLF*, *B4GALNT3* and *GPCPD1*, *P _discovery+replication_* <5E-8), and two additional novel loci with suggestive evidence of replication in all replication studies (*5q31* and *DLC1*, *P _replication_* <0.05). This allowed us to add additional evidence to our novel findings compared to previous replication results (*B4GALNT3* was successfully replicated at *P _discovery+replication_* <5E-8, and *5q31, HLF* and *GPCPD1* exhibited suggestive evidence of replication at *P _replication_* <0.05).**

**The sample sizes and association summary statistics in each study in the replication stage are shown in Supplementary Table 5. The total sample sizes of African and Hispanic ancestry populations are larger than the ones in the PAGE discovery analyses, but several loci did not show evidence of replication in these ancestrally matched samples. Potential heterogeneity within ancestral groups may contribute to the failure of replication. As we mentioned in the Discussion section (lines 392-395), the Hispanic participants in the current analysis came from various regions including Central and South America, Puerto Rico, and Mexico, and were grouped together when performing the association testing. Another contributor might be the potential GxE interactions that were not evaluated in our analyses. We added a sentence to the Discussion section (line 365) indicating that further investigation and successful replication of these ancestry-specific novel loci are needed.**

3. Conditional analyses. These were performed using individual level data in PAGE, and approximate conditioning with GCTA in ENGAGE, but what was the reference used for LD.

**Response: Thank you for the comment. Since all participants in ENGAGE were of European descent, the LD matrix was estimated from 9,345 European ancestry participants from the ARIC study that were available in PAGE. We added this information to the manuscript (lines 479-481).**

4. The authors mention testing for heterogeneity in effects due to ethnicity by testing for interaction with PCs in PAGE, but I couldn’t find any information in the methods about this, nor any plots of PCs to demonstrate that this was an appropriate analysis/interpretation. Overall, I found the methods very sparse, and much more detail is needed to allow others to repeat experiments.

**Response: We appreciate the reviewer’s comment and added more details to the Methods section (lines 493-499). In brief, interaction analyses were performed by including SNP×PC terms for all the first 10 PCs in the models when examining the potential heterogeneity of previous known loci in PAGE minority ancestry participants. Models with and without the interaction terms were then compared using the F-statistic, and an overall SNP×PC interaction *P* value (*P_SNP×PC_*) for each known variant was estimated, which indicated whether the additional variance explained by the interaction terms was statistically significant and represented effect modification driven by genetic ancestry.** **The *P_SNP×PC_* values for all previously reported variants are shown in Supplementary Table 7.**

5. FINEMAP analyses. My understanding from reading the methods is that FINEMAP with first applied within each ethnic group, and then the results combined across ethnic groups. However, there are no details provided for the LD reference used for these analyses, and the parameter settings used, such as number of causal variants in a region. It is also not clear to me that combining the posterior probabilities across ethnicities as described is appropriate, so some justification of this approach is needed.

**Response: Yes, FINEMAP was first applied within each ethnic group, namely African American, Hispanic and European ancestry groups, and then the results were combined across these three ancestral groups.** **LD references used for the African American, Hispanic and European ancestry groups were estimated using all African American participants genotyped on the MEGA array (N=17,324), all Hispanic participants genotyped on the MEGA array (N=22,244) and European ancestry participants from ARIC study (N=9,345). The number of causal variants in each region was set to 1 in FINEMAP. This information was added to the manuscript (lines 515-519).**

**We agree that the approach we used to combine the posterior probabilities is different from the commonly used one, which is first combining association results through meta-analysis and then generating posterior probabilities based on the meta-analysis results. In the current scenario,** **the sample size of European ancestry population from GLGC (N=188,577) is overwhelming compared to the ones of AA (N=17,641) and Hispanic (N=22,830) ancestry populations in PAGE. As a result, the meta-analysis results combining these three groups are mainly driven by GLGC results, which in turn introduces bias in the fine map analysis. The approach we used took into account both the association *P* value and the sample size of each group, which would be a better way to estimate the combined posterior probability for each variant. We added this information to the Methods section (lines 523-529).**

6. MTHFD2 locus. The authors highlight a SNP in strong LD with the index SNP, but it is not clear in which population group this LD is calculated? It would be more sensible to quote the relative posterior probability from the fine-mapping analysis, compared to the lead SNP – is it even in the 99% credible set? Similarly, I found listing SNPs that happened to have some interesting annotation that have r2>0.2 with an index SNP unimportant – again, it is not clear in which population the LD has been measured, or the relative posterior probabilities.

**Response: Thank you for your comments. The LD of the *MTHFD2* locus was calculated using all African American participants genotyped on the MEGA array (N=17,324) in PAGE since it was driven by African signals. For each of the nine novel loci, we added details on how LD matrix was estimated in the Methods section (lines 529-537).**

**We previously performed fine-map analyses only for the reported loci, but we now also did perform fine-map analyses for the nine novel loci according to the comments and presented the results in Supplementary Table 12. At the *MTHFD2* locus, the 99% credible set consisted of six variants, but the LD proxy (rs186506279) we mentioned ranked 7^th^ in the results. We deleted the sentence describing this variant in the Results section. Combining the functional annotation and fine-map results, we did observe a variant in the 99% credible set of the *B4GALNT3* locus (rs34019521, r^2^=0.85 with the lead variant), which overlapped with enhancer activity in liver and showed an Eigen PC phred score of 22.11 (≥17, functional). These results were added to the Results section (lines 311-315). In addition, we excluded SNPs that showed weak LD (0.2≤r2<0.4) with the top hits in Supplementary Table 14 according to the comment.**

7. Table 2. It would be useful to have: MAF for each ethnic-specific analysis; the final combined meta-analysis p-value across discovery and replication; p-values to two significant figures throughout.

**Response: Ethnic-specific MAFs in African American, Hispanic and European ancestry participants and the final *P* values of meta-analyzing discovery and replication studies are shown in Table 2. All *P* values are set to two significant figures throughout our manuscript.**
