# Supplementary material for: Minority-centric meta-analyses of blood lipid levels identify novel loci in the Population Architecture using Genomics and Epidemiology (PAGE) study
Source: PLoS Genet. 2020 Mar 30;16(3):e1008684. doi: 10.1371/journal.pgen.1008684 (PMC7145272; doi:10.1371/journal.pgen.1008684)
Supplement: Attachment — Submitted filename: PAGE_lipids_rebuttal_R2.docx [file pgen.1008684.s023.docx]

Reviewer #1: The authors have addressed most of my comments, and I believe the manuscript has been improved. However, I have a couple of outstanding comments...

1. I had not realised that the FINEMAP analysis had been performed assuming a single causal variant at each locus. Given this assumption, it is not clear why the authors have run FINEMAP, since this has been designed to allow for multiple causal variants at the locus. With a single causal variants, I believe that the LD reference is irrelevant, since no joint models with multiple SNPs are fitted. In theory, the association summary statistics for each ethnicity could just be used to calculate a Bayes' factor and posterior probability for each SNP. I think the methods should also state that the posterior probability calculation across ethnicities assumes that there is a single causal variant at the locus, and that this variant is the same in each ethnic group.

**Response: Thank you for the comment. We agree that the LD reference is irrelevant when setting the number of causal variants in each locus to 1 in FINEMAP. We added one sentence to the Methods section (line 523-524, page 23) indicating that the posterior probability calculation across ethnicities assumes that there is one single causal variant at each locus, and that this variant is the same in each ethnic group, and that consequently the FINEMAP results do not depend on the LD.**

2. Heterogeneity assessment using SNP x PC interaction effect. I like this way of assessing heterogeneity due to ancestry, but wasn't clear why 10 PCs were used. Please provide justification. Including 10 PCs (if they are not needed) will substantially reduce power to detect heterogeneity (since an extra degree of freedom is needed for each PC in the test).

**Response: Thank you for the comment. We used two criteria to determine how many PCs should be included in the analysis, namely reference population specificity and chromosome specificity, which were detailed in a previously published paper (PMID: 31217584).**

**In Figure 1 (Supplementary Figure 10 of the published paper, reproduced below), the first 10 PCs showed clear population specificity in the 1000 Genome Phase 3 reference samples. PC9 and PC10 were clearly African in origin in the reference data (top panel), although these two PCs did not vary tremendously within our PAGE study.**

**PCs which load uniformly across chromosomes are likely to represent ancestral populations, while PCs that load heavily onto specific chromosomes are frequently artifacts in the data. Results of a loading analysis are presented in Figure 2 (Supplementary Figure 11 of the published paper, reproduced below), which shows the correlation between SNP genotype and PC1 through PC20. PC1 through PC8 are clearly consistent across the genome, so their inclusion as covariables is justified. PC9 and PC10 posed a unique challenge. Population specificity in Figure 1 suggested that these two might be important in African populations, arguing for inclusion, but the chromosome loading results in Figure 2 suggested that they could be artifactual. In our published paper (PMID: 31217584), we conservatively chose to retain these two PCs in the analysis, and the marginal association analysis results with eight PCs were similar to the ones with 10 PCs.**

**We further calculated the F-statistic when including different numbers of PCs with a sample size of 30,000 (an approximation to the actual sample size of 33,063 in PAGE MEGA data). We set the probability of type 1 error when including all 10 PCs to 5%, and the probabilities of type 1 error when including one to nine PCs, respectively, are shown in Table 1 below. These probabilities are very conservative as they assume that there is not even ‘random heterogeneity’ for the irrelevant PCs. Thus, this table would suggest that our testing with 10 PCs rather than eight PCs would mean that we test at a level not lower than 3.6% if PC9 and PC10 show no random heterogeneity. If they do, the actual significant level is somewhere between 3.6% and 5% - so we are not reducing the power much.**

Figure 1. Standardized PCs by population.

After standardizing the ranges of PC1 through PC32, we plotted the value for each individual as a line. The top panel shows individuals within the 1000 Genome Phase 3 reference population (colors were coded by population, and PAGE study samples were coded grey). The bottom panel shows PAGE participants colored by their self-reported ancestry (reference populations were coded pink). This allows us to see the distribution of different race/ethnicity groups across the different PCs. For example, in the top panel we see orange lines at the outer ranges of the distribution, indicating that PC3 represents the spectrum of Native American ancestry, as orange denotes reference groups from the Americas.

Figure 2. Correlation between SNP genotype and the first 20 PCs by chromosome. Genomic position is shown on the X axis and the correlation between genotype and each PC is shown on the Y axis (ranging from 0 to 1).

Table 1. F-statistic with different numbers of PCs in 30,000 samples

|  | F-statistic | Type 1 error |
| --- | --- | --- |
| PC1 | 3.84 | 3.23E-5 |
| PC1-2 | 3.00 | 8.73E-4 |
| PC1-3 | 2.61 | 3.68E-3 |
| PC1-4 | 2.37 | 8.39E-3 |
| PC1-5 | 2.21 | 1.44E-2 |
| PC1-6 | 2.10 | 2.12E-2 |
| PC1-7 | 2.01 | 2.84E-2 |
| PC1-8 | 1.94 | 3.57E-2 |
| PC1-9 | 1.88 | 4.23E-2 |
| PC1-10 | 1.83 | 5.00E-2 |

3. Tables still do not have p-values presented to two significant figures - Table 2 has a mixture of two and three significant figures (e.g. 4.89E-5 is three significant figures, 0.47 is two significant figures). I think all tables (including supplementary could do with proof reading) - e.g. in ST16, the causal allele frequency of rs11782435 is 2022%?

**Response: Thank you for the comment. We have checked all tables (including supplementary tables) and made sure that all *P* values throughout the manuscript were presented to two significant figures and the numbers in each table are in the right format.**
